# Thermally Sprayed Functional Coatings and Multilayers: A Selection of Historical Applications and Potential Pathways for Future Innovation

**DOI:** 10.1007/s11666-023-01587-1

**Published:** 2023-04-27

**Authors:** Edward J. Gildersleeve, Robert Vaßen

**Affiliations:** Institute of Energy and Climate Research, Materials Synthesis and Processing (IEK-1), Forschungszentrum Jülich GmbH, 52425 Jülich, Germany

**Keywords:** aerospace, biomaterials, embedded sensors, energy conversion, environmental barrier coatings (EBCs), membranes, thermal barrier coatings (TBCs)

## Abstract

Thermal spray coatings are material systems with unique structures and properties that have enabled the growth and evolution of key modern technologies (i.e., gas turbines, structurally integrated components, etc.). The inherent nature of these sprayed coatings, such as their distinctive thermal and mechanical properties, has been a driving force for maintaining industrial interest. Despite these benefits and proven success in several fields, the adoption of thermal spray technology in new applications (i.e., clean energy conversion, semiconductor thermally sprayed materials, biomedical applications, etc.) at times, however, has been hindered. One possible cause could be the difficulty in concurrently maintaining coating design considerations while overcoming the complexities of the coatings and their fabrication. For instance, a coating designer must consider inherent property anisotropy, in-flight decomposition of molten material (i.e., loss of stoichiometry), and occasionally the formation of amorphous materials during deposition. It is surmisable for these challenges to increase the risk of adoption of thermal spray technology in new fields. Nevertheless, industries other than those already mentioned have benefited from taking on the risk of implementing thermal spray coatings in their infrastructure. Benefits can be quantified, for example, based on reduced manufacturing cost or enhanced component performance. In this overview paper, a historical presentation of the technological development of thermal spray coatings in several of these industries is presented. Additionally, emerging industries that have not yet attained this level of thermal spray maturation will also be discussed. Finally, where applicable, the utility and benefits of multilayer functional thermal spray coating designs will be demonstrated.

## Introduction

### Contemporary Growth of Thermal Spray Technology Based on Market-Driven Research and Innovation

Thermal spray technology has been fortunate to experience decades of continuous growth and adoption globally across a number of industries. In particular, the versatility, scalability, and prospective benefits from utilizing thermal spray coatings have proven to be some of the most attractive aspects of implementing thermal spray technology in many industrial sectors. In 2013, Dorfman and Sharma reported the global market for thermal spray technology to be roughly estimated at around $6.5 billion (Ref 1). Based on more recent market research, that number is estimated for 2020 to be around $7.6 billion, with a compound annual growth rate (CAGR) of around 7%. This would imply that by 2025, the thermal spray market can globally reach around $10.7 billion in net value (Ref 2).

In addition, based on these same market research reports, the thermal spray coatings market was experiencing continued healthy growth leading up to 2020. However, the growth from $6.5B in 2013 to $7.6B in 2020 is not reflective of the rate at which the market was growing within those years. Thus, this mild stagnation of market growth suggests a somewhat substantial event, which changed the overall trajectory of the market. Clearly, the socioeconomic impact of the COVID-19 pandemic was significant enough to result in a shift in the thermal spray market trajectory. Even now, in 2022, the estimated market value of thermal spray technology has not reached the levels that were anticipated back in 2019 (Ref 2).

**Fig. 1:**
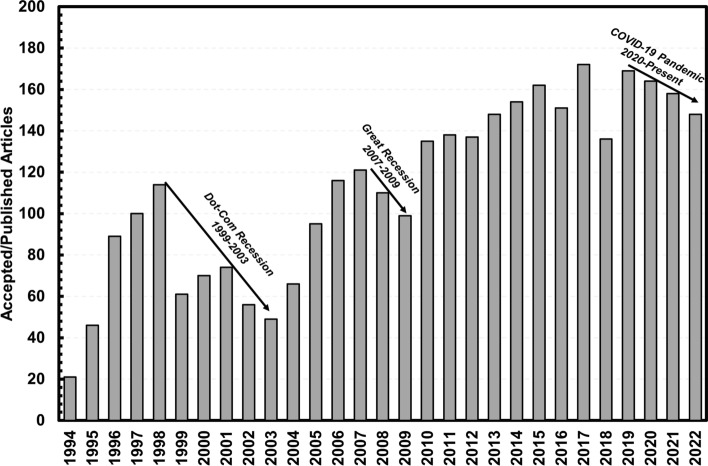
Accepted and/or published articles in the Journal of Thermal Spray Technology from 1994 to 2022 (Ref 3)

This economic market information becomes interesting when cross-examining with the available metrics of research in thermal spray technology concurrently. Figure 1(a) shows the publication metrics for the *Journal of Thermal Spray Technology* from 1994 to the present, specifically the total number of accepted or published articles in the journal for that specific calendar year (Ref 3). It is important to note that research and publications in thermal spray technology are not solely funneled through the *Journal of Thermal Spray Technology*; however, as this Journal has an exclusive focus to publishing research in thermal spray (as opposed to other journals), the data here are intended to give a more general overview of the status of thermal spray research activity over these years. From the figure, it is clear that some years underperformed relative to the overall trend of Journal growth. These fluctuations seemingly correlate with major global events i.e., the United States Recession(s) (1999-2001, 2007-2009) and the COVID-19 Global Pandemic. It is surmisable to consider that the trends in quantity of research published in the *Journal of Thermal Spray Technology* over these years can be reflective of thermal spray research in other journals as well. Therefore, one can conclude the economic thermal spray market and the research in the field are both swayed by major socioeconomic events, as suggested in the Figure. In other words, if the overall global market falters due to certain events, these aftershocks are felt in all areas of thermal spray technology. Therefore, before adoption in new and emerging markets can be considered, it becomes necessary to be cognizant of these interrelationships. Additionally, it is interesting to look at thermal spray coatings and how they have been utilized and researched to probe if it is possible to break this intertwined relationship. The focus for this overview is on understanding the progression of thermal spray technology in this context in so-called functional thermal spray coatings.

#### “Functional” Thermal Spray Coatings as Studied in Academia

From its earliest use cases, thermal spray coatings were traditionally considered to be a “protective” or “sacrificial” layers. Examples of this use of thermal spray include noble or active coatings for corrosion protection (i.e., ‘metallization’), coatings for wear resistance (i.e., ‘tribological coatings’), or coatings to protect against oxidation of superalloys (i.e., nickel-based alloy coatings) (Ref 4-7). However, at this time, if these coatings failed, the system(s) could still maintain themselves. More recently, thermal spray coatings are considered to have more specific “functions” that, if rendered useless, could inhibit or fully stop the processes they are enabling. An example of this would be in the case of coatings for protection in jet turbines, specifically those used to protect the components from ingestion of foreign debris. If the protective coating were to fail, the jet turbine can easily become clogged with ingested debris and subsequently fail prematurely. Still, the term ‘functional thermal spray coatings’ has historically been somewhat ambiguous and will be discussed further.

The first documented use of the explicit term ‘functional coating’ with thermal spray technology in the available public literature is credited to Alonso et al. in 1991. This study examines plasma-sprayed erosion-resistant Al_2_O_3_-based and cermet coatings for tribological applications on carbon-fiber epoxy composites (Ref 8). Also, in the early 1990s, Neale et al. at the United States Air Force compiled an interim report for thermally sprayed thermoplastic powder coatings for corrosion protection (Ref 9). In this report, there are specific ‘Functional Coating Performance Properties,’ which acts as a general list of the desired properties of the corrosion barrier coating. These ‘functional property requirements’ of the coating include high toughness, high resistance to salt fog and water, and resistance to environmental factors such as extreme heat and ultraviolet exposure. In 1994, J.W. Pickens at NASA stated in a study wherein wire arc-sprayed coatings were applied to fiber-composite drums: ‘if this cannot be solved by further control of the process conditions, the presence of a functional coating on the fiber…may be needed’ (Ref 10). Then again in 1998, FMJ van den Berge publishes in Advanced Materials & Processes: ‘the goal of all (these) thermal spray processes is to provide a functional coating that meets all the necessary requirements…’ (Ref 11).

From this point, the use of the term ‘functional coating’ in the public literature expands and goes on to cover a wide variety of meanings (Ref 12-16). Hereafter, this overview article chooses to define ‘functional coatings’ simply as coatings that do more than simply imparting slightly improved component lifetime, i.e., as a ‘sacrificial layer’. Rather, in this article, ‘functional coatings’ are considered as those which in some way provide a unique capability to the overall system that otherwise would not be present without the coating(s). In particular, the ‘functional coatings’ examined in this overview will cover a broad range of industries (well-established, evolving, and prospective). These examples are connected by a common theme of demonstrating published literature on coatings, which can provide the potential for thermal spray technology to break through to new industrial applications and fields. For further clarification of this overview’s definition of the term ‘functional coatings,’ an example of a coating transforming from ‘imparting improved lifetime’ to ‘functional’ will be shown here.

#### Thermal Spray Coatings—from ‘Protective’ to ‘Functional’ a Case Study of Thermal Barriers

One example of a thermal spray coating, which has transformed from ‘imparting improved lifetime’ to ‘functional’, is thermal barrier coatings (TBCs). Originally, TBCs were also considered to be a layer, which solely extends the lifetime of the turbine component, wherein the TBC functioned as a sacrificial entity. Original TBC applications utilized fully stabilized zirconia ceramics with stabilizers such as calcia, magnesia, and yttria (Ref 17-19). These coatings, while technically thermally insulative and effective in reducing the surface temperature of the underlying superalloy, had markedly poor long-term thermomechanical durability. However, because the TBCs at the time were, as mentioned, intended to be more ‘sacrificial’ than ‘functional,’ the low durability was accepted in order to gain the thermal insulation capacities. As the technological needs in the underlying gas turbine industry advanced, the temperature capabilities of turbine components advanced as well, which inevitably required the same evolution from the coating technologies.

Comparatively, TBCs now are significantly more robust and concurrently have much more reliable long-term durability metrics than their predecessors. As such, these modern TBCs fall under this overview’s definition of ‘functional’ as ‘existing beyond more than merely a sacrificial protective layer’. However, these improvements were driven by several catalysts set forth by both manufacturer needs and the global market. Specifically, the need for more power output (and later the need for more fuel-efficient electric power generation) drove these technological advancements. Turbine inlet temperatures were mandated to increase to improve the power output and fuel efficiency. As a consequence, the requirements of TBCs also changed from being ‘protective’ to more ‘functional.’ For instance, in order to combat the spallation induced by attack from molten ingested siliceous debris, TBCs could no longer exist merely as ‘sacrificial layers’ as they did in the late twentieth century. Rather, careful and deliberate concurrent research by materials scientists and surface engineering experts enabled the realization of evolved next-generation TBCs. These next-generation TBCs are strategically manufactured with materials that, for instance, have an affinity to chemically react with the molten debris and prevent long-term degradation. This is discussed in detail in the later sections.

**Fig. 2:**
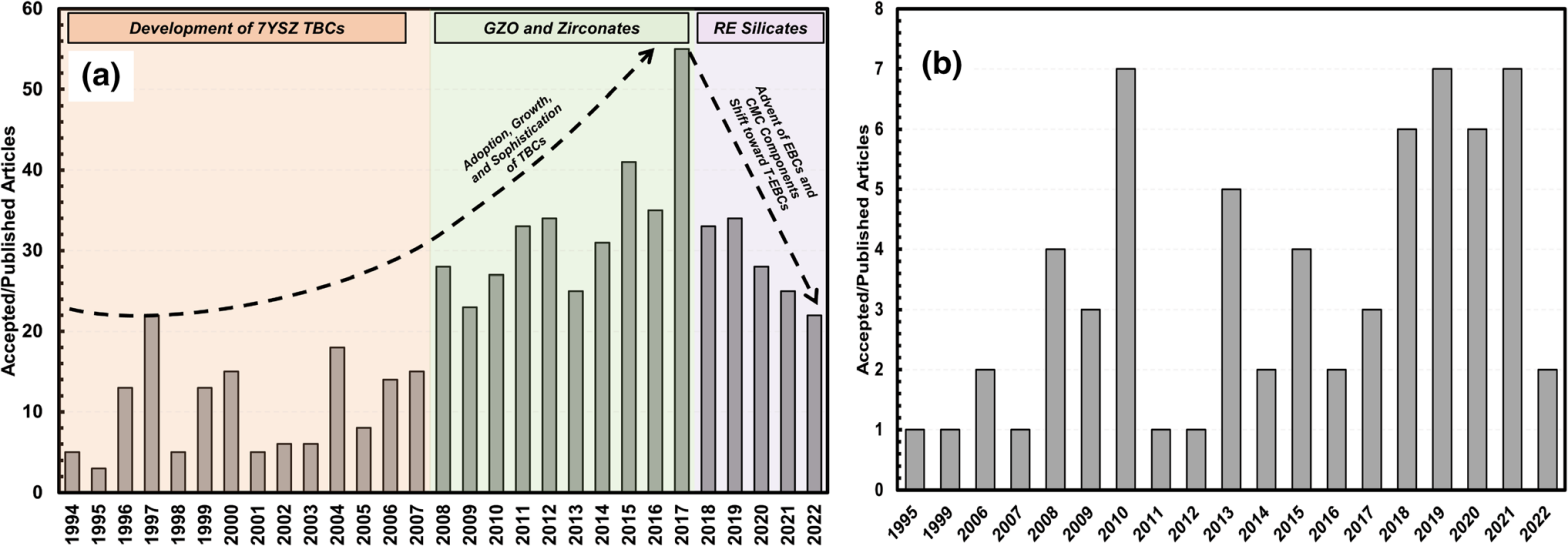
(a) Accepted or published articles in the Journal of Thermal Spray Technology which focus on ‘thermal barriers’ and (b) accepted or published articles in the Journal of Thermal Spray Technology which focus on ‘functional multilayers

One can find parallels in the technological progression of thermally sprayed TBCs by once more studying the trends in the published literature. Here again, publication metrics from the *Journal of Thermal Spray Technology* are used as an example from the same time period as shown in Fig. 1. Figure 2(a) shows the accepted or published articles in the Journal by year that specifically focus on ‘thermal barriers’ (Ref 3). It is important to once more note that the *Journal of Thermal Spray Technology* metrics are used as an illustrative or comparative tool, but not claimed to define the absolute number of published articles in TBCs. Nevertheless, one can argue based on the trends that research and the industry closely followed one another. As the industry and its needs grew, so did involvement and publications by researchers. For example, with the recent shift in turbine technology moving toward ceramic components and even higher turbine inlet temperatures, TBCs are being superseded by next-generation environmental barrier coatings (EBCs). Interestingly, the published research in the Figure for ‘thermal barriers’ specifically reflects this shift in technological interest.

#### Future Outlook for ‘Functional Thermal Spray Coatings’

It is evident when cross-comparing the growth of the thermal spray market with the growth of research and publications that the movements of both the global and sector-specific markets heavily sway the direction of growth in thermal spray technology. Whenever the market demanded improvements, research and innovation subsequently followed, as can be seen in the case study of TBCs. However, the converse to this implies that as market demand and value of thermal spray technology experience some regression, research in the field equivalently can be stunted (i.e., the shift in focus from TBCs to EBCs). This market-driven research and innovation approach has allowed thermal spray technology to sustain itself for more than one hundred years; however, the question remains: is this a maintainable course of action for the next one hundred years? When examining Fig. 2(b), the order of magnitude difference in volume of published literature and research in ‘functional thermal spray coatings’ as compared to more-established fields such as TBCs and EBCs becomes obvious. However, one can argue that this implies the thermal spray industry has an opportunity to diversify, pivot, and capitalize on the opportunity of exploring sprayed coatings for more functional applications. These ‘new and emerging’ applications and opportunities will be described in detail in this overview.

### New and Emerging Applications of Thermal Spray Technology—An Opportunity for Novel Coatings and Coating Design

As previously mentioned, many applications in thermal spray technology began (and some still maintain themselves) as single-layer (in some cases sacrificial) surface coating solutions applied to mitigate deleterious operating conditions. While advantageous (i.e., low-cost, easy to fabricate, versatile processing, etc.), this approach has some implicit disadvantages. As an example, single-layer thermal spray coatings can often be equivalently considered ‘single-use’ or ‘single-function’ coatings. An example of these single-use applications comes again from turbines, wherein single-layer coatings can commonly provide insulation from elevated temperatures or preventing ingression of corrosive media. However, in many industrial sectors, application needs are becoming increasingly diverse—for instance, the need for thermal insulation and protection against ingression of corrosive media simultaneously in jet turbines. In this context, the idea of monolithic coatings can be viewed as somewhat restrictive in regard to not being able to optimize the coating structure for all performance needs.

Researchers have in the past published work in response to this restrictive nature of monolithic single-layer coatings. For instance, functionally graded materials, (FGMs) were studied heavily in the 1990s and early 2000s (Ref 20-28). Yet, despite the volume of research, very few of such innovative technologies have been seen to permeate the commercial industry since their inception. One can consider, for instance, that perhaps the envisioned performance benefits did not outweigh the added complexity or assumed risk of adoption.

Again, looking at thermal barrier coatings for gas turbines, specifically land-based as an example, most TBCs are still applied as primarily single-layer, single-material (yttria-stabilized zirconia, YSZ) coating solutions in the industry. However, for the application technology to improve, it is implied that the coating technology must subsequently progress and innovate as well. After FGMs in the 1990s and 2000s, the concept of ‘multilayer’ thermal spray coatings, wherein discrete (microstructure, material, or both) layers have been integrated into one singular composite coating system has been tried and proven in recent literature for a variety of applications (Ref 29-35). Still, there is inertia in adopting new, more complex coating processes to facilitate performance gains. Figure 2(b) shows using the same *Journal of Thermal Spray Technology* metrics that even in research publications, the idea of ‘functional multilayers’ in thermal spray has not yet seen the same amount of traction. It therefore becomes important to attempt to somehow establish causal relationships that can better define the hesitance for industries to either adopt new, more advanced thermal spray coatings, or use the technology in general. In this overview, several emerging or evolving industrial fields will be studied, and a historical progression of the field(s) in the context of the role thermal spray coatings has had in the evolution of these technologies.

This paper has assimilated and categorized these historical progressions into five sub-categories: biomedical coating solutions, coating solutions for sensors and electronics, coating solutions for waste heat reclamation and energy production, coating solutions for gas sensing and separation, and coating solutions for extreme environments. Several common threads thematically connect the research and the coatings presented in this overview. First, as a general theme amongst the research assimilated here, in taking a historical approach from premiere publications to most recent advancements in the field, the progression from monolithic coatings to more complex multifunctional coatings can be seen in almost every case. In addition, by taking this historical approach, one can envision how multifunctional thermal spray coatings have the potential to enable technological advancements in new and emerging fields.

## Applications in the Biomedical Fields

### Multilayer Osteoconductive Bioceramic Coatings

Thermally sprayed coatings for biomedical applications have existed conceptually and in practice for several decades. One of the first mentions of using coated components for biomedical applications can be traced back to 1981—where De Groot alludes to the use of thin plasma-sprayed ceramic coatings comprised of hydroxyapatite (HA) atop metallic implants (specifically titanium metal/metal-alloys) (Ref 36, 37).

However, the inception of integrating plasma-sprayed HA into medical implants came only after originally utilizing and studying sintered ceramic HA materials. It was found that despite the significant bioactive compatibility of HA (discovered by Denissen, De Groot, and Jarcho (Ref 38-40)), there were substantial biomechanical integrity issues with the sintered HA ceramic components (Ref 41, 42). Specifically, despite high compressive yield strengths of ~ 700 MPa, and tensile yield strengths of ~ 250 MPa, the resistance to fatigue of these sintered HA ceramics was very low (Ref 43). In fact, it was found by de Groot that within only a few months, repeated tensile physiological loading would cause fatigue fracture of the sintered HA ceramics (Ref 42). Therefore, it was necessary to investigate and implement a more mechanically robust solution that in parallel possessed long-term biocompatibility—hence the benefits of plasma-sprayed HA drove the research forward.

**Fig. 3:**
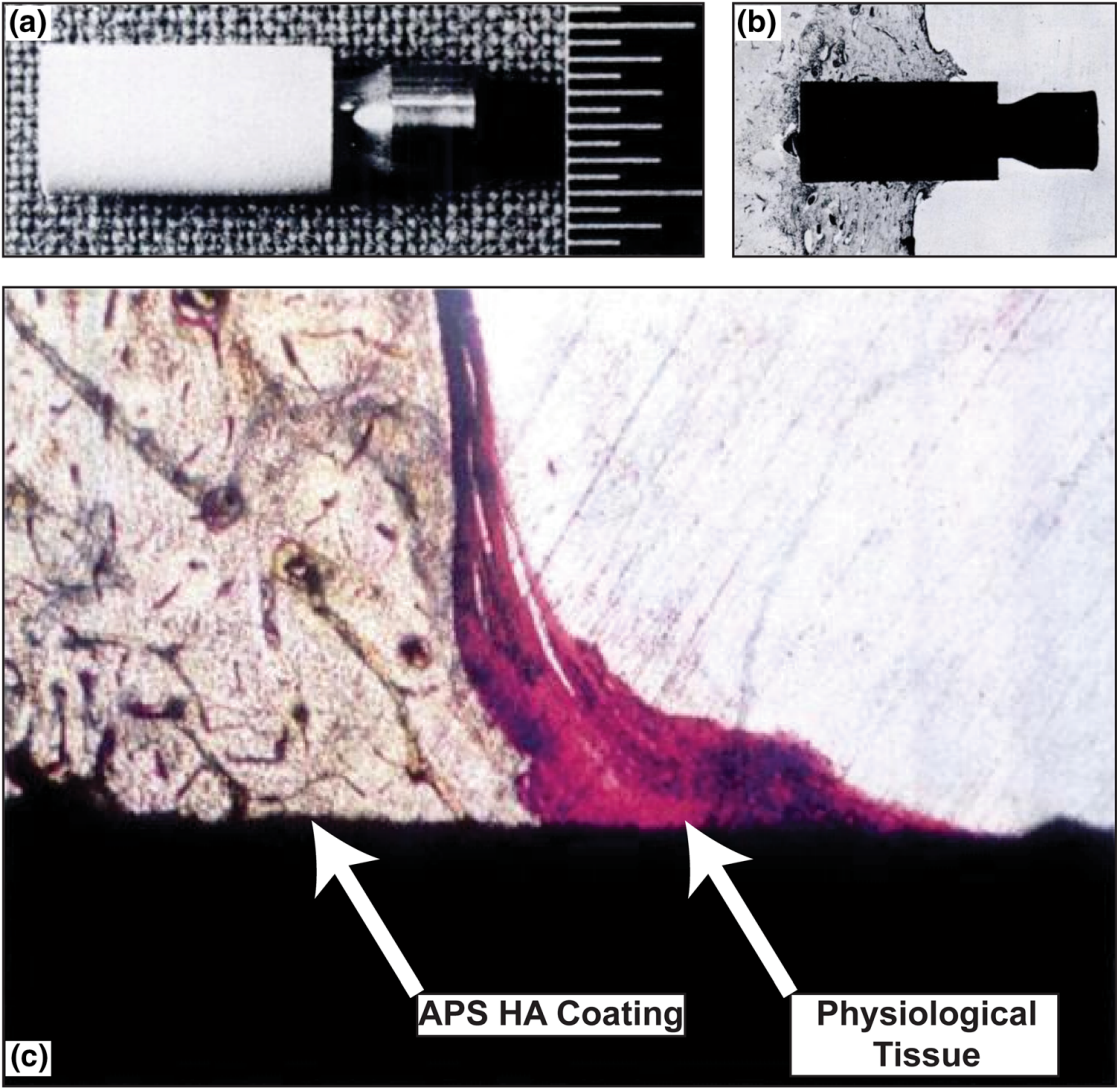
(a) Plasma-sprayed hydroxyapatite-coated implant installed in dog femur, the scale bars are 0.5 mm. (b) Histological section of an implanted zone showing the outline of the implant. (c) Zoomed-in histological section showing the integration of physiological tissue with the APS HA coating. Figure adapted from Geesink et al. (Ref 43). Used with permission of British Editorial Society of Bone & Joint Surgery, from Bonding of bone to apatite-coated implants, R.G. Geesink, K. de Groot, C.P. Klein, The Journal of Bone & Joint Surgery (British volume), volume 70, issue 1, 1988; permission conveyed though Copyright Clearance Center, Inc

While porous-metal-coated implants received approval by the United States Food and Drug Administration (FDA) in the 1990s, it was not until 2004 when plasma-sprayed HA coatings would receive explicit mention as a FDA-approved technology (Ref 44). For reference to a sense of scale in this application, in 2012, one company working exclusively on thermally sprayed biocompatible coatings, Medicoat AG, recorded a milestone of 500,000 thermal-spray-coated medical implants since its founding in 1989. Figure 3 shows a plasma-sprayed hydroxyapatite-coated implant used in a dog femur studied by Geesink et al. (Ref 43); the physiological bonding between canine tissue and the coating can be seen.

Given the history, plasma-sprayed HA can certainly be considered a relatively established technology that is used extensively in the biomedical field. The research that follows demonstrates areas for further improvement of the technology. Several disadvantageous aspects to the current state-of-the-art plasma-sprayed HA coatings have been identified in the literature, and they are as follows:

- Local substrate heating during deposition can cause undesirable phase transformations in the underlying titanium alloy ($\alpha \to \beta$)
- APS coatings are limited in their minimum thickness capabilities (i.e., it is difficult to spray coatings below 20 µm thickness). Most biomedical coating applications minimize coating thickness to minimize residual stresses and the propensity for delamination (as the residual stress and energy release rate for fracture both are proportional to the overall coating thickness (Ref 41, 45-48)
- APS HA coating performance is highly porosity-dependent. For osteoimplant coatings, a pore size > 75 µm is ideal for optimal cell in-growth (Ref 41, 49)
  - However, large pores can be sites for bacteria colonization
- Thermal decomposition of the material in-flight during deposition can be uncontrollable, and yield undesirable coatings from an osteoconductive point of view.

Given the diverse nature of weaknesses in these original coatings, it becomes challenging for researchers to address these shortcomings by only considering a single layer of coating material. For instance, making a highly porous plasma-sprayed HA coating may yield substantial gains in human cell integration with the coating. And yet, the resulting microstructure may be so mechanically weak that it is substantially more prone to delamination than its predecessors. Alternatively, one may focus on optimizing the as-sprayed stoichiometry of the plasma-sprayed HA coatings; however, in doing so, it may be impossible with the requisite spraying parameters to integrate the appropriate type of microstructure/porosity needed. In this regard, one can consider multilayer “functional” osteoconductive coatings as a pathway to improve the present state of the technology. The progression of research in this direction will be shown hereafter.

The concept of a functional multilayer bioceramic coating on its own is also not entirely new. There have been studies in the past wherein functionally graded materials are utilized to optimize plasma-sprayed HA coatings. Khor et al. show that by iteratively grading a plasma-sprayed HA coating through-thickness with varying increments of Ti-6Al-4V blended with HA, residual stresses can be mitigated (Ref 22). Likewise, Brossa et al. and Zheng et al. found that adding Ti to HA (either in solid-state blending of powder or by integrating a porous Ti bond-coat) can improve the adhesion of plasma-sprayed HA beyond what was then the state-of-the-art monolithic style of coatings described earlier (Ref 50, 51). Gruner also showed a similar adhesion-promoting structure of a Ti bond coat, a Ti-HA composite layer, followed by a HA topcoat fabricated by plasma spray, while also showing fast and stable bone fusion (Ref 52). In all cases, these framework-establishing multilayer bioceramic coating studies cite optimal adhesion results, yet they do not capture the long-term durability implications of such a structure. Chen et al. show that with cyclic fatigue, these HA coatings (monolithic and functionally graded) can undergo significant microstructural changes, which in turn harm the coatings’ abilities to resist degradation and subsequent exposure of the titanium substrate to physiological media (Ref 53).

Clearly, the available FGM coating solutions in the literature are explicitly directed toward improving overall long-term coating adhesion. However, they often do not consider the physiological interactions that occur when the coating is introduced into the human body. As an example, suppose a coating is highly conducive to promoting bone integration. In that case, the consequence is that the coating becomes fully consumed by the osseointegration process, leading to eventual exposure of the titanium component to physiological media. Once exposed, the metal component can release metal ions into the body, which can in turn lead to long-term complications (Ref 54-56). To mitigate this, a more robust coating strategy would be required—one that promotes long-term adhesion while also preventing the degradations associated with coating consumption by osseointegration.

Singh et al. demonstrated one such concept of a multilayer, in this case also functionally graded, bioceramic coating (Ref 57, 58). In this study, coatings are introduced, which are substantially thicker than the state-of-the-art plasma-sprayed HA coatings, but consequentially have more functionality. To compensate for the 2-3 times thicker coatings, a ceramic bond coat layer is introduced. In this case, Singh et al. show that introducing a plasma-sprayed titania (TiO_2_) layer at the titanium—bioceramic interface can not only mitigate mismatch strains due to thermal expansion, but also promote long-term adhesion (Ref 57, 58). The authors in this study reiterate that the functionality of a plasma-sprayed HA coating is at least twofold: the surface interactions between coating and human tissue, and the long-term degradation in physiological media of the entire component.

**Fig. 4:**
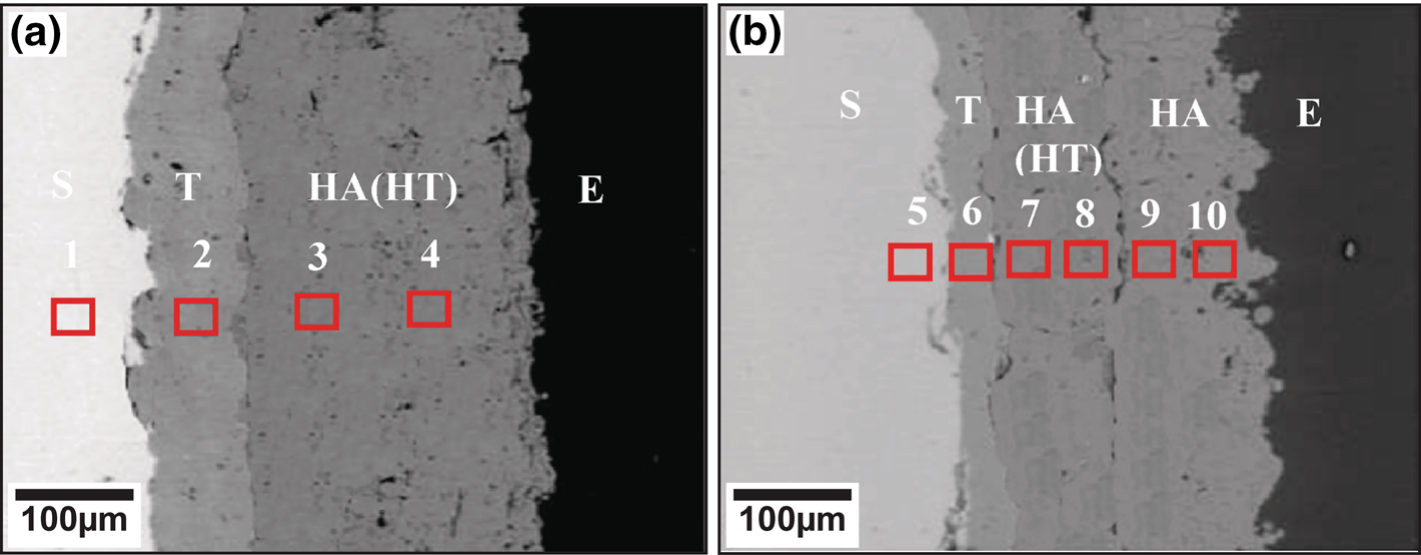
Multilayer thermally sprayed implant coatings as proposed by Singh et al. (Ref 58). (a) Multilayer coating with a TiO_2_ bond coat (#2), heat-treated (HT) hydroxyapatite (HA) intermediary coating (#3), and plasma-sprayed amorphous HA coating (#4). (b) Functionally-graded version of the multilayer coating as proposed by (a). Figure adapted from Ref 58. Reprinted from Ceramics International, volume 47, issue 7, Jarnail Singh, Sukhpal Singh Chatha, Hazoor Singh, Synthesis and characterization of plasma sprayed functional gradient bioceramic coating for medical implant applications, pages 9143-9155, Copyright 2021, with permission from Elsevier

At the surface of the multilayer coating proposed by Singh et al., highly amorphous plasma-sprayed HA is integrated—which has been seen to promote faster osseointegration and human cell growth (Ref 57, 58). Figure 4 shows two examples of the multilayer coatings proposed by Singh et al. (Ref 58). However, again the consequence of this improved osseointegration is the long-term dissolution of the material into the physiological media. It has been reported that the dissolution rate of a HA coating is as high as 60 µm within four years (Ref 58, 59). Clearly, if highly amorphous coatings dissolve at even more rapid rates, the mitigation strategy would be to prevent complete coating dissolution by maintaining coating crystallinity. On the other hand, the goal of any implant device is rapid physiological acceptance. Clearly, the diversity of needs in this application makes it evident that a single plasma-sprayed HA coating would be incredibly challenged to maintain osteoconductivity and long-term durability simultaneously, due to the interplay between coating crystallinity and osseointegration performance. Thus, the solution proposed by Singh et al. for the functional multilayer bioceramic include using plasma-sprayed and then heat-treated HA as an intermediate layer—resulting in a highly crystalline inner coating, while on the outermost layer, plasma-sprayed amorphous HA is introduced for enhanced osteoconduction. The net result of this effort is a coating, which has improved adhesion, optimized osteoconductive properties, and long-term durability.

### Multilayer Germicidal Coatings for Combating Bacterial and Viral Transmissions

While the integration of thermal spray hydroxyapatite for orthopedic implants has undergone almost thirty continuous years of research, development and innovation, the study of surface coating technologies for bactericidal or viricidal applications has comparatively seen less attention. Historically, the onset of research in utilizing thermal spray technology for this application can be seen as somewhat exploratory in nature, with the goal to develop some kind of surface coating system that can provide some extent of bactericidal functionality. The more recent surge in research in this field comes from two catalysts: the growing number of implant complications (i.e., bacterial infections) and the global COVID-19 pandemic.

#### Preliminary Studies on Thermally Sprayed Antimicrobial Coatings

The advent of studying surface functionalization using thermal spray for antibacterial and antiviral purposes can be traced back in the public literature to around the mid/late 2000s. In 2009, Jeffery et al. showed that the photocatalytic properties of TiO_2_ can be obtained in thermal spray deposits. Specifically, the high velocity oxy-fuel (HVOF) process was used to fabricate nanostructured TiO_2_ coatings for germicidal functionality (Ref 60). This early result showed that these first-trial TiO_2_ coatings were capable of destroying up to 25% of* Pseudomonas aeruginosa* bacteria within the first 120 min of exposure. Subsequently that same year, George et al. showed that introducing a small amount of copper into the TiO_2_ coating matrix significantly improved the bactericidal capabilities of the coatings under similar testing conditions (Ref 61). However, it was also determined in these early studies that the in-flight decomposition of TiO_2_ particles resulted in a deleterious phase change from anatase to rutile—which resulted in reduced photocatalytic activity (Ref 60, 61). In 2013, Ctibor et al. at the Institute of Plasma Physics in Czech Republic showed that photocatalytic activity of TiO_2_ coatings fabricated by thermal spray processes can, however, be recovered through spray process optimization, thereby implying that there was indeed a potential use for these coatings in future applications (Ref 62).

#### Limitations of HA-Coated Implants—Bactericidal Composite HA Coatings

Around the same time, studies were being published that identified complications and limitations in HA-coated orthopedic implants. In 2010, a review by Jafari MD et al. listed post-op bacterial infections as the leading cause for failure of most total hip arthroplasty (THA) procedures between 2000 and 2007 (Ref 63). Due to the porous and microcracked nature of plasma-sprayed coatings, these bioceramics were highly effective in promoting bone integration, but equivalently susceptible to the adhesion and formation of various bacteria colonies within the microstructure (Ref 64). Because of this issue of long-term development of THA bacterial infections, a new facet of research in surface-coated implants was developed—focused on the improvement of the plasma-sprayed HA/Ti composite coatings to resist colonization and growth of bacteria.

In 2008, Song et al. showed for HA coatings (formed with micro-arc oxidation (MAO) processes) that the inclusion of silver particles within the HA coating provided antibacterial benefits (Ref 65). In 2012, this finding was reproduced by Roy et al. using thermal spray deposition techniques to produce RF plasma-sprayed HA composite coatings with Ag dopant inclusions (achieved by solid-state synthesis of the powder prior to deposition) (Ref 66). In both cases, it was found that the inclusion of Ag within the HA coating allowed for some degree of antibacterial benefit. However, there are implications of long-term biotoxicity with any amount of Ag particle inclusions, which inhibited the large-scale adoption of this approach. More recently, Pham et al. in 2022 showed that by utilizing the same approach, but substituting Ag with Ga, substantial antimicrobial properties can be achieved for implant coatings. This approach was inspired by using a material, which is significantly more accepted in the medical community as having low intrinsic biotoxicity (Ref 64).

#### Thermally Sprayed Germicidal Surface Functionalization: An Overview of Modern Findings

In the last five years, there has been a substantial surge in research on utilizing thermal spray coatings as functional surfaces for germicidal purposes. This most recent surge was of course largely fueled by the global COVID-19 pandemic and the desire to fabricate functional surface coatings, which limit the growth and survival rate of bacteria and viruses. The following section presents an overview of several key findings from this field.

Copper and its alloys are among the earliest materials discovered to have germicidal properties. Use of copper for hygienic and antimicrobial purposes dates back to the early Egyptians as well as the Greeks, Romans, and Arabic civilizations which recognized the highly beneficial properties of metallic copper against certain infections and diseases (Ref 67). While seemingly it would be sensible to manufacture furniture, hardware, etc. in public spaces out of metallic copper, the manufacture and processing of copper sheet metal into these form-factors is labor-intensive and cost-ineffective in a modern industrial society (Ref 68). More commonly, stainless steels are used for such components in public spaces. However, the disadvantages of stainless steel in this context are it possesses no resistance to bacterial growth and in fact can promote growth and colonization of bacteria in localized areas (i.e., in scratches, surface defects, etc.) (Ref 68). Therefore, research has been focused on utilizing surface coating technologies, such as thermal spray, to cost-effectively implement antibacterial materials onto stainless-steel surfaces already in public spaces.

**Fig. 5:**
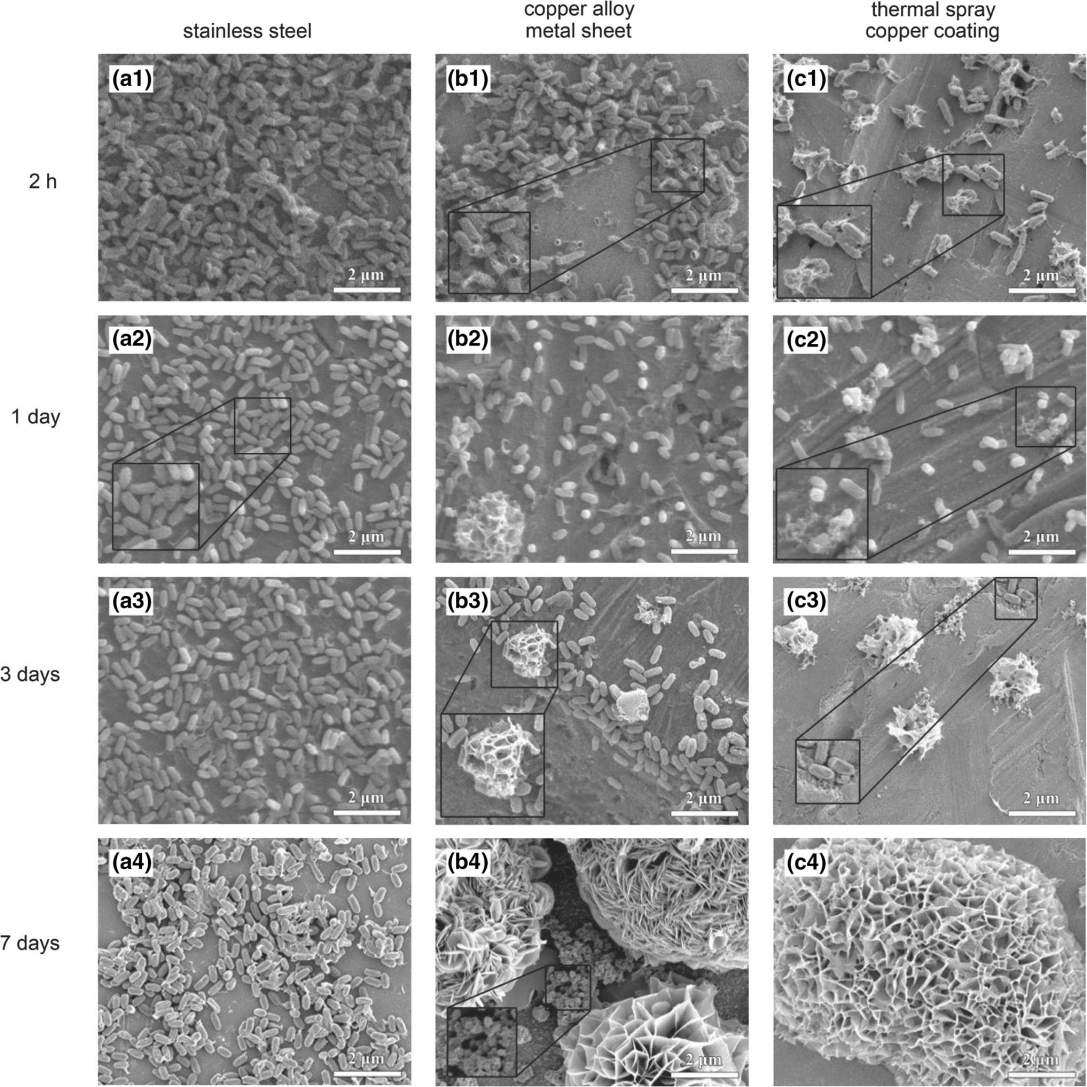
SEM micrographs of Bacillus Subtilis spores on top of (left) stainless steel alloy, (middle) copper alloy sheet metal, (right) copper alloy sprayed on steel alloy by twin wire arc spray. The separation and conversion of spores to nanoflower structures on copper alloys over the same time period are shown (Ref 68). Used with permission of Canadian Science Publishing, from Sporicidal efficacy of thermal-sprayed copper alloy coating, R. Shafaghi, J. Mostaghimi, V. Pershin, M. Ringuette, Canadian Journal of Microbiology, volume 63, issue 5, 2017; permission conveyed though Copyright Clearance Center, Inc

In 2017, Shafaghi et al. showed that by thermally spraying CuZnNi alloy using the twin wire arc (TWA) process, similar bactericidal mechanisms can be observed as achieved with CuZnNi alloy sheet metal (Ref 68). The bactericidal effects of the TWA coating are seen clearly in Fig. 5, wherein the conversion of bacterial spores to nanoflower-like structures is comparable between copper alloy sheet metal and the same composition TWA coating. In 2018, Jahani et al. reiterated and recognized the need for functionalized surface coatings to facilitate the development of antibacterial surfaces in modern society. Specifically, the authors in this article refer to thermal spray technology as having one of the most versatile and advantageous processing routes for future development of these functional surfaces—due to process flexibility, versatility, and scalability/cost-effectiveness (Ref 69).

The advent of the COVID-19 pandemic brought the need for antimicrobial surfaces from a ‘topic of interest’ to a ‘topic of urgent need’. In 2020, Harold and Corrine Michels published a perspective article in *Advanced Materials and Processes* illustrating the need to implement antimicrobial surfaces in common public spaces. In particular, the benefits gained by retrofitting of a public space with copper components were shown (Ref 70). Given the currently expanding volume of research on germicidal and virucidal surface coatings, especially those coatings formed by thermal spray technology, it is surmisable to consider there may be opportunities present with multilayer coating technology. However, at this time, the literature has not shown any specific examples of this.

## Applications in Sensors and Electronics

As mentioned in the introduction, one of the most attractive elements of thermal spray technology is the versatility of its scope of application. Materials selection and compatibility in thermal spray is seemingly infinite and more often is only limited by powder/feedstock synthesis capabilities. There are few technological examples that capitalized on this technological and materials versatility as significantly as thermally sprayed sensors and electronics. This section will show the growth and progression as documented in the public literature of alternative electronics manufacturing methodologies enabled by thermal spray processing.

### Early History of Thermally Sprayed Sensors and Electronics

The concept behind thermally sprayed sensors and electronics on its own traces back several decades. As early as 1970, Harris et al. studied Air Plasma Sprayed (APS) polycrystalline Ferrite coatings for their applicability in microwave integrated circuits (MICs) (Ref 71). In this case, APS was chosen among other materials processing routes at the time because film thickness requirements were greater than or equal to ~ 125 µm. The coatings were sprayed onto dielectric substrates, and it was found that with proper post-processing heat treatments, the magnetic and microwave properties of the systems were found to be comparable to that of bulk materials (Ref 71).

Around the same time, researchers in the Imperial College, London, studied spray-formed resistors, deposited again by APS. In this case, the work by J.C. Anderson et al. and Smith et al. illustrated to the scientific community the feasibility of using APS for depositing thick-film resistors (Ref 72, 73). It was determined by Anderson et al. that a mixed-oxide composition of 55% NiO // 45% Fe_3_O_4_ was among the most ideal candidates for a thermally sprayed resistor—as it yielded the lowest temperature coefficient of resistance (TCR) (Ref 73). From here, the work was continued by Smith in 1975 to evaluate the plasma processing effects on thermally sprayed NiO-Fe_3_O_4_ resistors sprayed on glass insulative substrates (Ref 72). These resistors were sprayed by blending two powders together volumetrically and then feeding through a single feed line. The resistors were evaluated based on both TCR and an ancillary parameter known as the third harmonic index (THI), which, loosely defined, is a measure of the projected longevity/durability of the resistor. From this initial work, it was determined that the chaotic lamellar microstructure of the thermally sprayed deposits was contributed to poor THI values as compared to conventional screen-printed or fired thick-film resistors. However, the authors did note the advantageous nature of depositing in a layer-by-layer fashion, wherein resistance/conductance of the sprayed device could be monitored in real time during fabrication to optimize the properties (Ref 72). The work by Smith et al. was continued in 1980, and it was determined that optimization of TCR was heavily dependent on the matching of particle sizes of the two materials prior to blending (Ref 74). Additionally, thickness effects and the effect of volumetric content of insulating phase on resistivity of the sprayed films were also studied.

### Toward Industrial Applications—Mask and Deposit Spraying

**Fig. 6:**
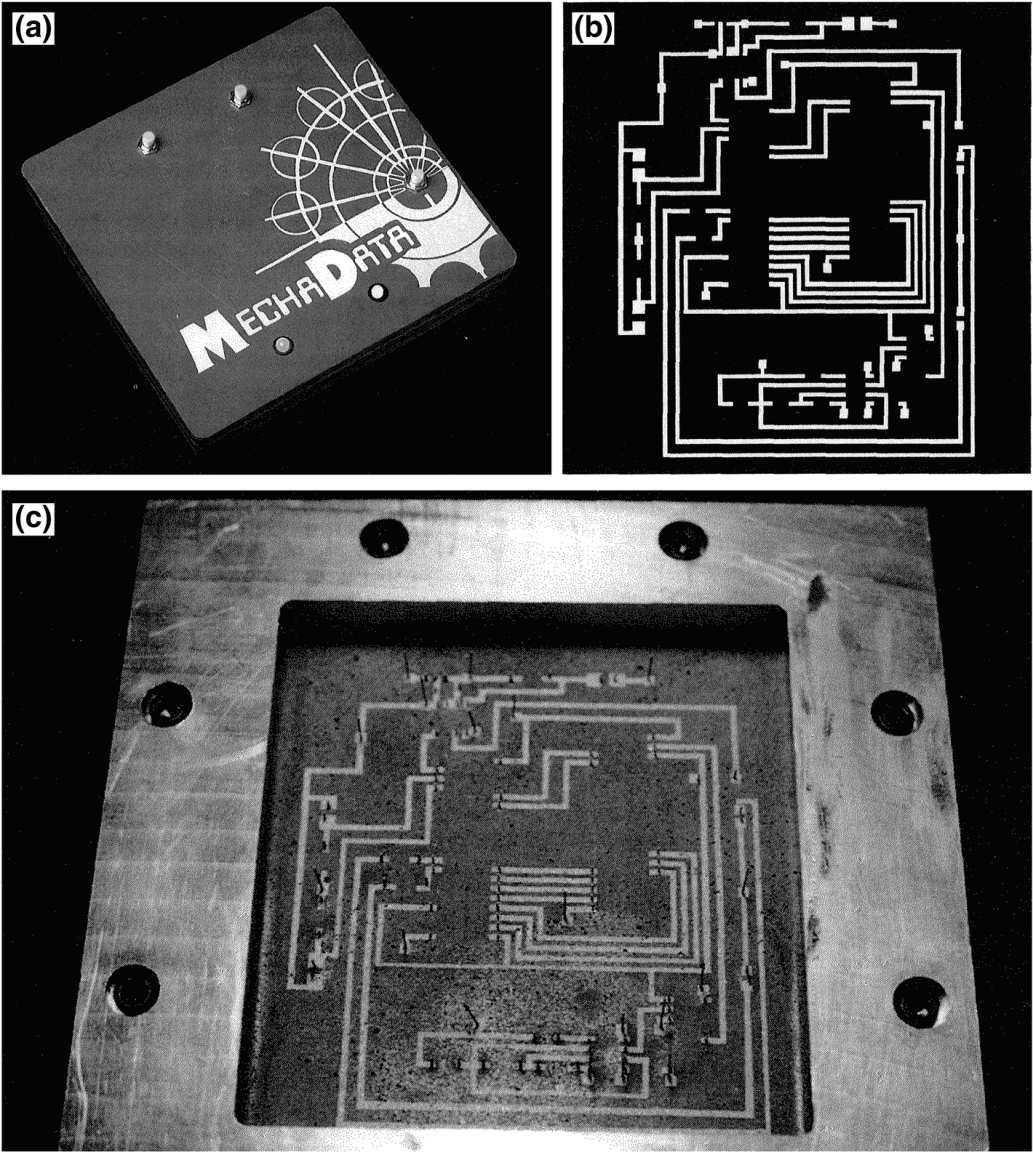
Mask-and-deposit thermally sprayed microcircuit as demonstrated by Beck et al. (Ref 77). (a) Final packaged microcomputer. (b) Computer-generated model of the first layer of microcircuitry. (c) First layer of microcircuitry deposited by plasma spray deposition as-modeled. Figure adapted from Ref 77

One can argue that once it was established that thermal spray technology possessed unique processing capabilities in the field of micro-/meso-electronics, the logical next stage of development in the application was to explore the ability to ‘pattern’ the spray deposits in particular locations, as is done in lithographic processes. Weiss et al. from Carnegie Mellon University showed the proof of concept of this processing methodology—dubbed the MD^*^ (Mask then Deposit) System (Ref 75, 76). With this methodology, 3D-CAD models for the ‘negative’ pattern were developed, allowing the machining of spray masks. Then after the deposition of each layer, the masks are removed, and new masks are applied until the device is completed. The method proposed by Carnegie Mellon was then utilized in 1992 by Beck et al. to manufacture a fully functional integrated micro-circuit (Ref 77). This device is shown in Fig. 6.

While an important milestone in the history of thermal spray technology’s role in micro-/meso-electronics and sensors, the MD^*^ fabrication process had several significant disadvantages. Most notably, the MD^*^ process was incredibly cumbersome and time-consuming. On a large-scale industrial production floor, it would be impractical to implement masking, mask-removal, and re-masking for tens of layers per device. Further to this point, in 1994, Merz et al. at Carnegie Mellon University made the decision to stray away from thermal spray technology entirely (Ref 78). The authors cited concerns over adhesion, bonding quality, and the aggressive nature of molten droplet deposition with respect to the masking technology associated with thermal spray. The proposed resolution in this work was to develop a hybridized process dubbed ‘microcasting,’ which is also a droplet-based deposition process, but employs material droplets 10-100 × larger and significantly slower/more controllable than what is seen in plasma spray deposition in order to promote local substrate remelting for enhanced layer adhesion (Ref 78).

Besides micro-computers, a number of other devices were fabricated using the MD^*^ method in the mid-1990s. Fasching et al. describe the efforts made to fabricate over 140 type T, E, and J thermocouples using iron, nichrome, and Cu-Ni alloys as the feedstock materials (Ref 79). In this case, instead of APS, the thermocouples were fabricated using the twin wire arc (TWA) process. All thermocouples made with this process were found to be comparable to commercially available systems. In addition, a combined humidity sensor/heater was also fabricated with the MD^*^ process in this study. This system comprised of (in order, through-thickness, bottom-to-top) an Al_2_O_3_ insulator layer, Zn-embedded heating elements (~ 50 µm thick × ~ 1.5 mm wide), another Al_2_O_3_ insulator, a Zn-integrated humidity sensor, a porous Al_2_O_3_ moisture-absorbing layer, and sprayed thermocouples. At the time of Fasching et al.’s publication, the feasibility of the sensor was not studied; however, it was noted by the authors that there are possibilities of the molten metal droplets impregnating the porous underlying insulators and causing short-circuits (Ref 79). Nevertheless, these initial studies and publications helped in establishing thermal spray technology as a feasible processing route for the fabrication of electronics, albeit with some needed improvements.

### Addressing the Shortcomings of MD^*^ Spray—Direct-write thermal spray

The aforementioned research can be considered to have provided the framework to enable thermal spray technology to penetrate into the global electronics market. However, the shortcomings of these original approaches—cumbersome spray methodologies, complicated tooling, long concept-to-device lead times, all had to be overcome in order to successfully implement thermal spray technology in this market. Still, in the early 2000s, thermal spray technology was utilized in microelectronics and sensor research via a mask-and-deposit methodology, with Sampath et al. showing a multilayer MnZn ferrite-based thermally sprayed inductor and a patent by Klassen et al. in 2001 demonstrating mask-and-deposit thermal spray technology for fabricating a multilayer electrical interconnection device (Ref 80, 81). In 2000, Sampath et al. list the deficiencies inhibiting the growth of thermal spray technology in this field to: lack of fundamental understanding of process–material–property relationships; absence of diagnostic tools to evaluate and optimize the process; insufficient process control; and limited personnel with expertise in the field (Ref 80). A breakthrough that pushed the technology forward was the development of thermal spray direct write technology (Ref 82, 83).

**Fig. 7:**
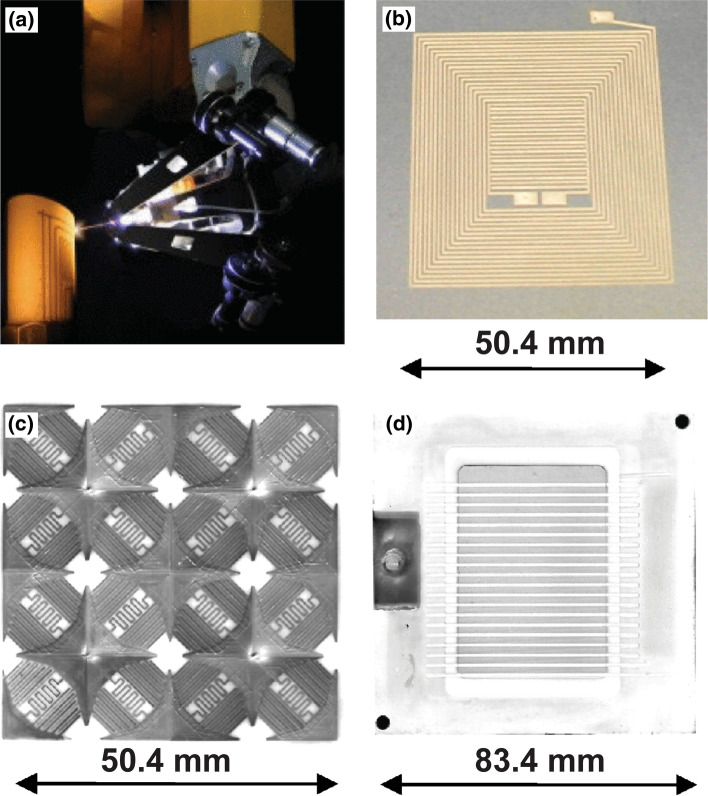
(a) Direct-write thermal spraying a custom circuit on a curved surface as shown by Gallagher et al. (Ref 93). Copyright 2013 IEEE. Reprinted, with permission, from M.W. Gallagher, W. C. Smith and D. C. Malocha, An integrated SAW sensor with direct write antenna, 2013 Joint European Frequency and Time Forum & International Frequency Control Symposium (EFTF/IFC), pp. 450-453. (b-d) an L-C circuit, Frequency selective surface, and multilayer inductor with ferrite core all fabricated by direct-write thermal spraying as shown by Gouldstone et al. (Ref 92). Reprinted from C. Gouldstone, J. Gutleber, W. Smith, Direct‐Write Strain and Temperature Sensors for Harsh Environments, AIP Conference Proceedings, volume 894, issue 1, pp. 949-956, Copyright 2007, with permission from AIP Publishing

In 2003, researchers at SUNY Stony Brook filed a US Patent on the *Method and Apparatus for Fine Feature Spray Deposition*. Here, a completely novel spray torch/collimator/dynamic aperture system was introduced to the community (Ref 82, 83). The advantages of this aperture system would be that the spray plume is no longer unbounded upon exit from the torch nozzle. Rather, the feature size of the spray deposit could be restricted or controlled by said aperture. This novel system also would allow for deposition of fine materials at incredibly low and precise feeding rates to overcome a stated concern in the previous literature—the stacking and bonding quality of lamellae during spray deposition (Ref 75, 76, 78). A photograph of direct-write thermal spray and several exemplar devices fabricated by the technology are shown in Fig. 7.

Some of the earliest devices to be demonstrated from the direct-write thermal spray technology include thermocouples and strain gauges. Longtin et al. in 2004 demonstrated the proof of concept of this direct-write thermal spray apparatus by showing sprayed features, which had far exceeded the dimensions and capabilities of its predecessors (Ref 84). Line thicknesses of $\ge$ 20 µm and line widths of $\le$ 200 µm were now possible with this technology (Ref 82-84). Furthermore, as Longtin et al. demonstrated in this study, it had become possible to modulate the line width and thickness in situ during spraying, which is helpful for devices such as strain gauges—wherein the sensor should have significantly smaller dimensions than the lead contact points (Ref 84). Not only did the performance of the strain gauges meet the same standards as commercially available hardware in this study, but the flexibility of direct-write thermal spray was again shown by demonstrating these sensors can be embedded easily within a functional system (Ref 85-87). In addition, these early studies demonstrated the feasibility and competitive performance of spray-formed Type-E and Type-K thermocouples.

Along with thermocouples and strain gauges, DWTS also proved to have the potential to fabricating more sophisticated sensors. Ahn et al. showed by using DWTS that spinel-based humidity sensors could be fabricated using this technology. This stack of four discrete coating layers, all around 30 µm in thickness and between 85 and 95% dense, proved to respond to changes in humidity by exhibiting changes in device impedance in a monotonic manner (Ref 88). This was associated with the increase in surface electrical conductivity due to water adsorption within the embedded spinel dielectric layer (Ref 88). DWTS also enabled thermocouples to be embedded within coatings at discrete positions. Gutleber et al. and Theophilou et al. demonstrated that by doing this, it is possible to create a “heat flux sensor” out of multiple embedded thermocouple junctions through-thickness (Ref 89, 90). Using a DWTS heat flux sensor in the high heat-flux laser rig at NASA-GRC, the incident heat flux through a thermal barrier-coated superalloy button was measured to be around 75 W/cm^2^ (Ref 89, 90).

Direct-write thermal spray was also advantageous in that it was an additive process that could be combined with subtractive processes to generate complex sensor configurations. For instance, DWTS was coupled with ultrafast laser micromachining (LMM, i.e., using a femtosecond UV laser) to preferentially machine away certain sprayed areas. This allowed DWTS to be repeated after the LMM process and enabled flexibility and new possibilities in sensor configurations.

Gouldstone et al. showed that by combining DWTS with LMM, one can achieve high-definition features with even finer resolution than DWTS alone (Ref 91). In this work, strain gauges were deposited onto a ceramic ‘blanket,’ then laser micromachined to isolate the individual conductive lines from the remainder of the system. This effectively lengthened the conduction pathway and, in turn, allowed the as-deposited resistance values of the DWTS grid to be on the order of commercially available foil strain gauges at the time (Ref 91). One additional advantage to the DWTS/LMM strain gauge system was that the inherent nature of bonding between sensor, ceramic blanket, and substrate was superior to the commercially available technologies of polymer adhesive systems, and the work by Gouldstone et al. demonstrated the durability and utility of such systems in extreme conditions (Ref 91).

Later, Gouldstone et al. would show examples of LC circuits, frequency selective surface sensors, and multilayer inductors with ferrite cores, all fabricated using this high-definition direct-write process (Ref 92). As the technology of high-definition DWTS further matured, published work in the public domain becomes limited. However, the growth and development of the technology into a commercial product via Mesoscribe™ Technologies, Inc., are duly recognized. To this day, Mesoscribe™ Technologies (now under the CVD Materials Corporation Umbrella) still continue to fabricate direct-write sensors and devices in the industrial aerospace, power generation, satellite, and defense sectors and is actively working on major US federal grants. In 2013, Gallagher et al. reported the use of DWTS to build metallic antennae on top of surface acoustic wave sensor substrates (Ref 93)—a feat previously achieved with only limited success (Ref 94).

From this point onward, publicly available literature referencing any form of thermally sprayed sensor technology is highly limited. This can be surmised to be due to the maturation and commercialization of the technology. The most recent research activities in the meso-electronics field focus mainly on using thermally sprayed coatings to enable new functionality in gas sensors. This concept will be overviewed in a subsequent section.

## Applications in Waste Heat Reclamation and Energy Production

The prior section showed the flexibility and versatility of the thermal spray process to deposit and fabricate non-conventional materials for unique applications. Whereas most thermal spray applications serve as protective coatings against wear, corrosion, or extreme environments, the prior section showed utility in utilizing the technology to fabricate sophisticated and integrated multilayer, multi-material systems in novel and innovative ways to enable new functionalities and technological breakthroughs. In this section, an equivalently novel application opportunity for thermal spray technology in enabling waste heat reclamation will be shown.

### Thermoelectric Materials—Background

One topic of fundamental research that has received constant attention by the materials and manufacturing community is the prospect of reclaiming wasted/lost heat as usable energy. The production of energy or electrical current from heating two dissimilar metals due to the migration of charge-carriers was observed by Thomas Johann Seebeck in 1821 and is known as the Seebeck effect, defined by the Seebeck coefficient $\alpha \left[\mu V/K\right]$ (Ref 95-97). Modern research in this field is devoted mainly toward utilizing the Seebeck effect to generate electricity from waste heat via ‘thermoelectric generators’.

The quest for renewable and cleaner sources of energy can be considered as the catalyst to researching thermoelectric materials and generators for the last several decades. The fundamentals of thermoelectric generators, the Seebeck effect, and the physics behind the phenomena have been well-defined in many other studies (Ref 96, 97). Briefly, thermoelectric generators and materials are classified in this context based on the thermoelectric figure of merit $Z={\alpha }^{2}\sigma /\lambda$, where $\sigma$ is the electrical conductivity and $\lambda$ is the lattice thermal conductivity (often replaced with the experimentally measured thermal conductivity of the material) (Ref 98). The figure of merit can be made dimensionless by measuring the value at a given temperature $ZT$. A higher figure of merit generally implies that such a material will be highly efficient in converting waste heat to electrical energy. Therefore, most research in this field is focused on optimizing the figure of merit either by synthesis of new materials or improvements in processing.

From a materials design perspective, metallic materials are governed by the Weidmann–Franz Lorenz law, which states that the ratio of thermal conductivity to electrical conductivity must remain constant (Ref 99). Thus, metallic materials generally are poor thermoelectric materials. This leaves the remaining material candidates of choice to be either oxides or semiconductors (or semiconducting oxides). Nonetheless, to this day, optimization of the figure of merit of any prospective thermoelectric material is still challenging, as generally speaking $\alpha$ and $\sigma$ do not change linearly with one another.

Further complicating the implementation of thermoelectric materials in industrial systems is the presently available thermoelectric generators (hereafter, TEGs) that are limited in their operational capacities. Generally, TEGs are classified and categorized based on their available temperature range of operation. So-called high-temperature TEGs operate at temperatures $\ge$ 800 K and are phase-stable over prolonged use. Examples of this include the Si-Ge alloy, which has been used in extraterrestrial applications as a TEG for decades (Ref 96, 97). On the other hand, ‘low-temperature TEGs are limited to operate at temperatures $\le$ 300 K, because they either undergo deleterious phase transformations at higher temperatures, or in some cases can volatilize, leading to overall loss of Seebeck performance (Ref 96, 97). One such example of an alloy in this case is bismuth telluride Bi_2_Te_3_. Current low-temperature TEGs are limited in use as either the material itself or the material processing route can be toxic, owing to limited adoption of the commercially available technology on a large scale (Ref 96, 97).

To that end, for TEGs to become widely adopted, a number of conditions must be met:

- The technology must be low cost and use readily available safe materials
- The technology must be easy to produce/fabricate, and production must be reproducible
- The technology must exhibit exceptional durability over long-term use
- The technology must have as much conversion efficiency as possible without compromising the above points

Current state-of-the-art TEGs are fabricated by conventional materials processing methods such as hot isostatic pressing (HIP) and spark plasma sintering (SPS) (Ref 100-104). The processing methodologies of current TEGs consequentially do not meet the above criteria. Assembly is usually done by hand and can be highly variable, and long-term durability is questionable as the adhesive strength between thermoelectric legs, interconnects, and the substrate(s) can be poor (Ref 105, 106). However, this processing methodology has enabled researchers to effectively study new and emerging materials for their thermoelectric performance. For instance, Tang et al. showed a substantially high Seebeck coefficient for TiO_2-x_ reduced single crystal synthesized materials (Ref 107); additionally, Mesaritis et al. demonstrated by using waste silicon dust from photovoltaic manufacturing that high-$ZT$ magnesium silicide (Mg_2_Si) could be fabricated (Ref 108). These methodologies, however, are limited in their scope of industrial adaptation because their fabrication routes do not meet the aforementioned criteria.

Therefore, thermoelectric technology is in need of a breakthrough processing route, which allows for these shortcomings to be solved. The following subsections will describe the role thermal spray technology has had in contributing to presenting a potential alternative processing route for future TEGs.

### Thermally Sprayed Thermoelectric Generators—An Overview

#### Preliminary Studies—Thermally Sprayed Silicides

In 1996, an international collaborative research group between Germany and the USA (comprised of members of DLR, RWTH-Aachen, and Drexel University) headed by Jürgen Schilz presented at the 15th International Conference on Thermoelectrics the feasibility of plasma spray-forming thermoelectric materials (Ref 109). In this proceeding, the authors note that the limiting steps between thermoelectrics and large-scale industrial adoption are the implementation of cheap, non-toxic materials and a processing method, which consistently, easily, and reliably can fabricate the TEG in a single manufacturing step. The authors point to the plasma spray process as a potential candidate to overcome these limitations (Ref 109).

The work by Schilz et al. demonstrates iron silicide (FeSi_2_) as a candidate material compatible with the plasma spray process. Iron silicide is a unique metal alloy, which possesses a semiconducting $\beta$ phase. This $\beta$ phase can be doped with cobalt or aluminum to elicit *n*- or *p*-type semiconducting behavior, respectively (Ref 110, 111). In this work, FeSi_2_ was sprayed using APS, vacuum plasma spray (VPS), and shrouded plasma spray (called SPS at the time). When FeSi_2_ is plasma-sprayed, the rapid solidification process yields mostly metallic $\alpha$ phase after the coatings are deposited (Ref 109). Schilz et al. revealed that this $\alpha$ phase can be transformed with post-spray heat treatments to $\beta$ phase. Furthermore, it was also determined that the thermal diffusivity of the sprayed coating (directly related to its thermal conductivity $\lambda$ from Sect. "Thermoelectric Materials—Background") approaches values, which are obtainable by bulk sintering of the material (Ref 109). From this preliminary study, it was also observed that the $ZT$ of the APS FeSi_2_ was around 0.014 in the as-deposited state and decreased by almost 100% after heat treatment at 800 °C for 50 h (Ref 109). Nevertheless, this result was still significant as it showed that the $ZT$ of the APS coating could rival that of the hot-pressed sample (0.014 versus 0.017)—showing the feasibility of APS as a thermoelectric processing method with further optimizations (Ref 109). These results and their ancillary studies were reiterated in an overview article by Bach et al. at the University of Dortmund in Germany to be an enabling set of results that allow for innovative material applications via thermal spray in the electronic materials industry (Ref 112).

More work on plasma-sprayed FeSi_2_ followed after these initial studies. In 1998, Tsutsumi et al. published in the proceedings of the 17th International Conference on Thermoelectrics showing their results on plasma-sprayed FeSi_2_ materials (Ref 113). In this case, rather than using shrouded-air plasma spray, the group used controlled atmosphere plasma spraying (CAPS) to further reduce oxidation and decomposition of the material. Furthermore, Tsutsumi’s group also was able to develop cobalt-doped FeSi_2_ feedstock material—at two different dopant levels—to improve the semiconducting behavior of FeSi_2_. Despite these efforts, the $ZT$ achieved by this study was still comparable to what was achieved in 1996. Interestingly however, this group was able to experimentally observe the anisotropic nature of the Seebeck coefficient of spray-formed thermoelectric materials—which was surmised to be due to the heterogeneity of the microstructure (Ref 113). Ueno et al. complimented this work and showed for the 5 at.%-cobalt-doped FeSi_2_ formed by CAPS, the $ZT$ value could exceed the value of the hot-pressed analog (Ref 114). Furthermore, with as much as a 300 °C temperature difference between the hot and cold side of a TEG made with 5%Co-FeSi_2_, 50 mV of voltage could be generated (Ref 114). At the same time, Schilz et al. characterized *p*- and *n*-doped FeSi_2_ by cobalt and aluminum, respectively, formed via shrouded plasma spray and found that the thermoelectric properties of these two compounds had the potential to exceed the bulk-pressed specimen (Ref 115).

In 1999, Huang et al. followed this work by spraying the same 5%Co-FeSi_2_ with both CAPS and high-velocity oxy-fuel (HVOF) spray using the JP5000 torch (Ref 116). It was determined the HVOF-sprayed FeSi_2_ thermoelectrics possessed a starkly different microstructure as compared to the CAPS variants. In HVOF, the lamellae in the as-sprayed state were visible, however, after heat-treatment to crystallize and transform the coatings to semiconducting $\beta$-phase, the lamellae disappeared, and the coatings became nominally denser. These unique features, however, did not yield a significant benefit in thermoelectric performance, as the $ZT$ was still on the same order of magnitude as what was determined in the 1998 study by CAPS and hot pressing (Ref 116).

While these initial studies on thermally sprayed FeSi_2_ provided the framework for the efficacy of thermal spray-forming thermoelectric materials, the thermoelectric properties obtained were still not competitive enough to contemporary devices/materials. To some extent, the consensus was that the thermal conductivity of sprayed thermoelectric materials was still not low enough to meet the envisioned potential of what the thermal spray processes could offer. This high through-thickness thermal conductivity mandated higher device layer thicknesses to impart the proper through-thickness thermal gradients for optimal output power. Lugscheider et al. in 1999 presented work on 10-mm-thick FeSi_2_ thermoelectrics formed by shrouded plasma spray using an SG-100 torch (Ref 117). These large thicknesses were chosen as a means to mitigate the inherently high thermal conductivity and produce coatings, which could induce a sufficient temperature gradient in a thermoelectric device (Ref 117). Furthermore, Lugscheider et al. showed in this study that again the flexibility of the thermal spray processes by producing graded structures. In this case, it was shown that the atomic dopant composition of cobalt could be modulated through-thickness to create graded TEGs easily with one manufacturing step.

From this point, the consensus among researchers in thermally sprayed iron disilicide semiconductors for thermoelectric materials essentially orbited around the following general points:

- The flexibility of the thermal spray processes to produce thick films at reasonable rates for minimal manufacturing costs is an attractive alternative to what is commercially available by sintering, etc.
- Thermal spray processing provides the opportunity to yield unique microstructures, chemistries, stoichiometries, metastable phases, etc., which may benefit the thermoelectric performance
- In all cases, thermally sprayed iron disilicide must be annealed after deposition, as the coating primarily consists of the metallic $\alpha$ phase, and the semiconducting $\beta$ phase is required to obtain thermoelectric properties
- The post-deposition heat treatment can, in some cases, remove some of the lamellar structure of thermally sprayed systems, which in turn can increase the thermal conductivity of the system and harm the overall thermoelectric performance

Clearly, the benefits of the thermal spray process were well-recognized by researchers for providing an alternative cost-effective method of manufacturing thermoelectric systems. At the same time, new thermoelectric semiconductors were also being considered for fabrication using thermal spray processes—for instance the deep space thermoelectric Si-Ge alloy as well as magnesium silicide Mg_2_Si (Ref 118). Unfortunately, in both studies the thermal conductivity was not measured, and it is not possible to extract the $ZT$ of these materials. Furthermore, the Mg_2_Si studied in Ref 119 while considered as a candidate for thermal spray, was excluded to studying materials formed by bulk synthesis.

While these alternative semiconductors (i.e., Mg_2_Si) for thermoelectric power generation were an attractive possibility, there were significant concerns based on what was observed from these initial studies with FeSi_2_ that the thermal spray process would cause undue stoichiometric and chemical shifts to even more unstable materials. This would to some extent be justified by the work done on thermally sprayed silicides for thermoelectric devices at Stony Brook in the mid-2010s. In 2013, Longtin et al. discusses the poor feasibility of thermally sprayed iron disilicide due to its inherently low $ZT$, yet cites magnesium silicide Mg_2_Si as a potential candidate material (Ref 120). However, the $ZT$ values of plasma-sprayed Mg_2_Si achieved by this study are almost two orders of magnitude lower than what was observed in the previous studies on iron disilicide (Ref 120). Semi-quantitative XRD analysis of these coatings revealed that there was significant oxidation in the APS coatings. Furthermore, the XRD spectra revealed the Mg_2_Si powder decomposed during deposition to form free Mg and free Si, which would explain the poor thermoelectric capabilities of the material. Interestingly, the Mg_2_Si sprayed by vacuum plasma spray (VPS) did not yield much free magnesium nor free silicon; however, this sample was not characterized for its thermoelectric properties in the study. In 2014, Fu et al. showed two new Mg_2_Si APS coatings; however, coating neither showed much marked improvement from the coatings studied in 2013 in terms of $ZT$ or thermoelectric performance (Ref 121).

#### Contemporary Research in Thermally Sprayed Thermoelectrics—Semiconducting Oxides

Around the same time when the limitations of the thermal spray process capabilities for semiconducting silicides were becoming apparent, the thermoelectric research community as a whole was simultaneously shifting away from these materials. This is because despite any inherent thermoelectric advantages that silicides (or even tellurides and skutterudites) may possess in the as-fabricated state, there are significant durability concerns for these materials. In a thermoelectric device application, the thermoelectric material must be exposed to moderately elevated temperatures and be environmentally robust. Skomedal et al. showed in 2013 that Mg_2_Si under thermal loading in air can dissociate and oxidize heavily (Ref 122). To this point, one study showed that by spraying the Mg_2_Si using VPS, the Seebeck coefficient could increase by an order of magnitude as compared to other coating processes showing the material’s thermoelectric sensitivity to oxidation (Ref 123). Likewise, the deep-space silicon–germanium thermoelectric alloys are known to sublimate under extreme environments (Ref 96, 97, 124). Thus, the general shift in thermoelectric research toward semiconducting oxides was in part for the sake of enhanced robustness of the materials.

Some of the advantages of using oxide materials for thermoelectric applications include: they can be nontoxic; they are generally chemically inert; they often are resistant to high/extreme temperatures; and they can be envisioned to have a long durability in extreme. Originally, the thermoelectric research community was unable to consider ceramic oxides in their research due to the inherently low electrical conductivity of these materials. The discovery of NaxCoO_2_ in 1997 as a strong thermoelectric candidate material, however, fueled the onset of research in so-called semiconducting oxide thermoelectrics (Ref 125, 126). Since then, layered cobalt oxide-based structured crystals have been studied extensively in the thermoelectric community and have been observed to have impressive $ZT$ values of unity or larger (Ref 127, 128). The research done by these groups on establishing the framework for feasibility of layered cobaltites in thermoelectric power generation later made its way to be researched by the thermal spray community.

In 2007, a research team of Sodeoka et al. between AIST Japan and Germany demonstrated one of the first thermoelectric oxide devices fabricated by plasma spray processing (Ref 129). This device was comprised of *p*-type (Ca, Bi)_3_Co_4_O_9_ and n-type (Sr, Y)TiO_3-x_, made by synthesizing feedstock material through solid-state synthesis methods then fabricating the coatings using CAPS. One advantage of using oxide materials is that the thickness requirements to get a sufficient temperature drop between hot and cold sides of the TEG can be substantially less than what was observed for the silicides. In this study, the calcium-bismuth cobaltite and strontium-yttrium titanate were sprayed only to 1.5 and 0.8 mm thickness, respectively (Ref 129). However, despite these inherent advantages, the thermoelectric performance (as characterized by the $ZT$ calculation done for this overview’s compilation of thermal spray coating $ZT$ data) showed no benefit to any of the previous thermally sprayed thermoelectric studies. In the case of the *p*-type calcium-bismuth cobaltite, the $ZT$ was comparable to what was obtained in the 1990s by the iron silicides. By comparison, the *n*-type strontium-yttrium titanate performed an order of magnitude worse than the calcium-bismuth cobaltite (Ref 129).

From this point, there is a noticeable ten-year gap in the available public literature between 2007 and 2017 wherein seemingly no articles are published on the use of thermal spray technology to form these next-generation oxide thermoelectric materials. It is surmisable to consider the performance benefits up until this point were not promising enough to merit further research on thermally sprayed TEGs. Nevertheless, following Sodeoka’s study, Lee et al. at Stony Brook University showed one of the most marked improvements from the past literature performance data in thermally sprayed TEGs. In this study, the group shows the feasibility of TiO_2_ as a thermoelectric material (Ref 130). TiO_2_ was chosen here following the framework set forth by the work of Tsuyomoto et al. and others. From Tsuyomoto, TiO_2_ exhibits unique semiconducting properties when it is reduced to TiO_2-x_. In fact, this study showed by reducing anatase TiO_2_ in a hydrogen atmosphere to TiO_1.94_, it is possible to achieve a peak Seebeck coefficient of − 518 µV/K and electrical conductivity of 1.9 × 10^3^ S/m, with a $ZT$ of 0.195 at 70 °C (Ref 131).

The approach adopted by Lee et al. was to utilize the unique nature of the air plasma spray process and capitalize on this enhanced thermoelectric performance of reduced TiO_2-x_ simultaneously. To do so, TEG devices were fabricated on ferritic stainless steel substrates using a similar mask-and-deposit approach as described in Sect. "Applications in Sensors and Electronics". From these results, some of the highest thermoelectric performance numbers reported for any thermally sprayed thermoelectric material were demonstrated. For the plasma-sprayed TiO_2-x_ material, a $ZT$ of ~ 0.13 at 750 K was achieved—a full order of magnitude higher than any of the other $ZT$ values reported for coatings at any temperature to this point in time (Ref 130). Clearly, the advantages of sub-stoichiometric fabrication of TiO_2_ from plasma spraying were demonstrated in this result. Additionally, to its benefit, the TiO_2-x_ was fabricated from commercially available thermal spray feedstock powder—which cannot be said about any other thermoelectric sprayed material to this point in time.

**Fig. 8:**
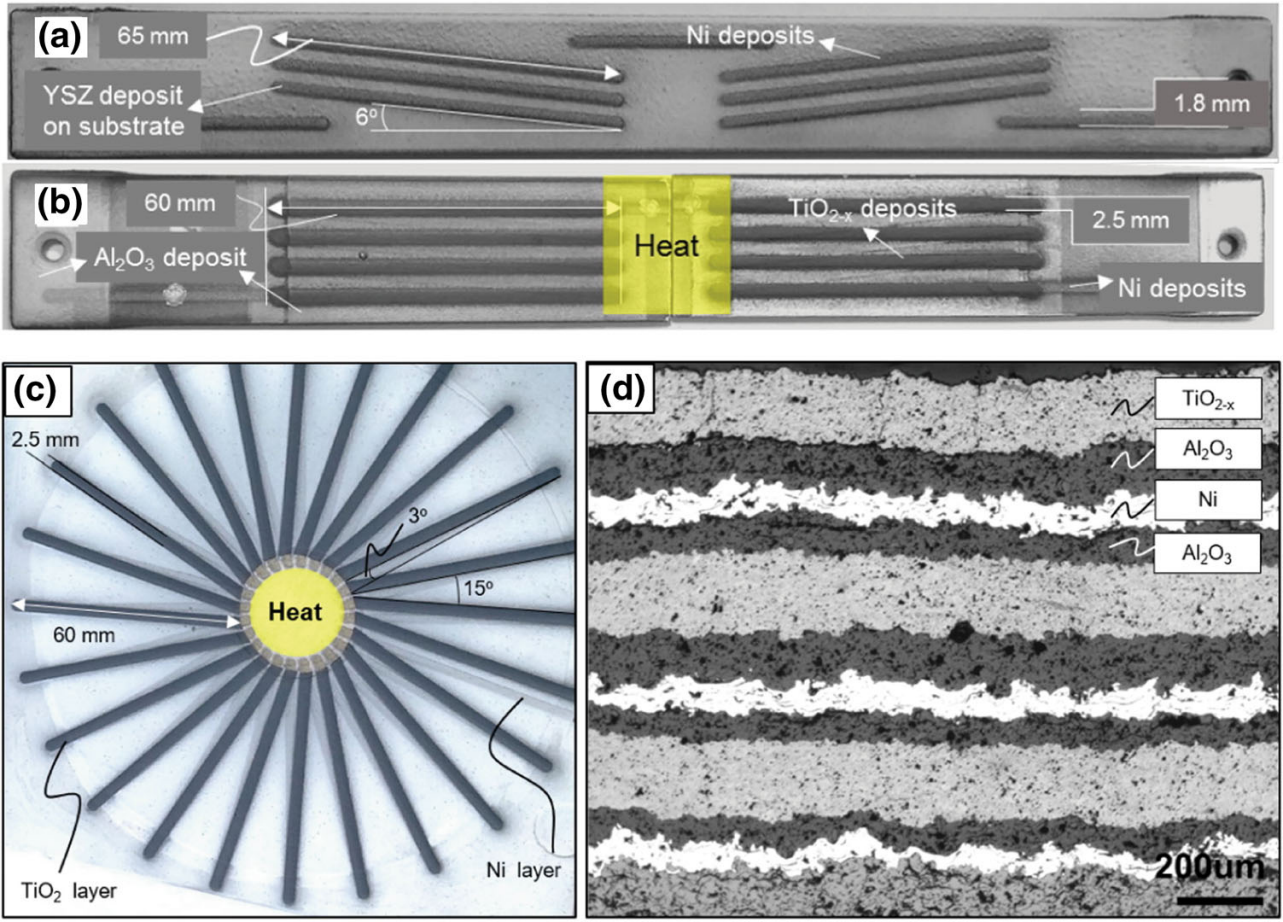
(a, b) Simple thermoelectric elements sprayed onto a 228 × 25.4 mm ferritic steel plate. (a) The first layer of Ni electrodes deposited on a yttria-stabilized zirconia insulative layer. (b) The subsequent layers of Al_2_O_3_ and TiO_2_ sprayed atop the Ni deposits. (c, d) show the more complex star-configuration thermoelectric generator formed entirely by Air Plasma Spray. Figure adapted from Ref 130. Reprinted from Applied Energy, vol. 192, Hwasoo Lee, Ramachandran Chidambaram Seshadri, Su Jung Han, Sanjay Sampath, TiO_2−X_ based thermoelectric generators enabled by additive and layered manufacturing, pp. 24-32, Copyright 2017, with permission from Elsevier

Lee et al. in the same study also demonstrated plasma-sprayed lithium-cobalt-oxide Li_x_Co_3-x_O_4_ (synthesized in-house) to have an equivalently impressive $ZT$ of 0.163 (Ref 130). For comparison in contemporary $ZT$ values, Song et al. in 2019 were able to achieve $ZT$ values of 0.18 at 1000 K for Ag-doped LiCoO_2_ formed by spark plasma sintering (Ref 132). Due to limited spray feedstock availability, and the goal of demonstrating fabrication of a thermoelectric device by thermal spray using only commercial and cost-effective processing routes, Lee et al. chose nickel as the n-type leg for all TEG devices. Nevertheless, the feasibility and applicability of multilayered thermal spray engineering and science for fabricating real thermoelectric generators were shown in this work (Ref 130). The thermoelectric devices—both in simple configuration and the more sophisticated star-configuration—are shown in Fig. 8.

Following this initial work, Lee et al. went on to study more specifically the processing space and flexibility in available thermoelectric properties when plasma-spraying commercially available TiO_2_ powders. From this work, a unique plasma spraying condition was found, which did not utilize secondary hydrogen gas, and yielded a $ZT$ of around 0.13, whereas all other TiO_2-x_ coatings in this study were generally an order of magnitude lower in $ZT$ (Ref 133). Lee et al. followed this work by studying APS calcium cobaltite—in this case Ca_2_Co_2_O_5_, following on the promising results obtained on a single-crystal Ca_2_Co_2_O_5_ material obtained by Funahashi et al. (Ref 127, 134). While the as-sprayed $ZT$ values were not substantial, after annealing one of the coatings showed one of the highest $ZT$ values (at 600 °C) of any thermally sprayed thermoelectric at 0.237 (Ref 134). Then, Caliari et al. took these two concepts of plasma-sprayed TiO_2-x_ and Ca_2_Co_2_O_5_ and integrated them into a new all-oxide thermoelectric device. The results showed that the device performance, under optimal microstructural and spray pattern tuning, could be competitive with other all-oxide thermoelectric generators that have been reported in the literature (Ref 135).

**Fig. 9:**
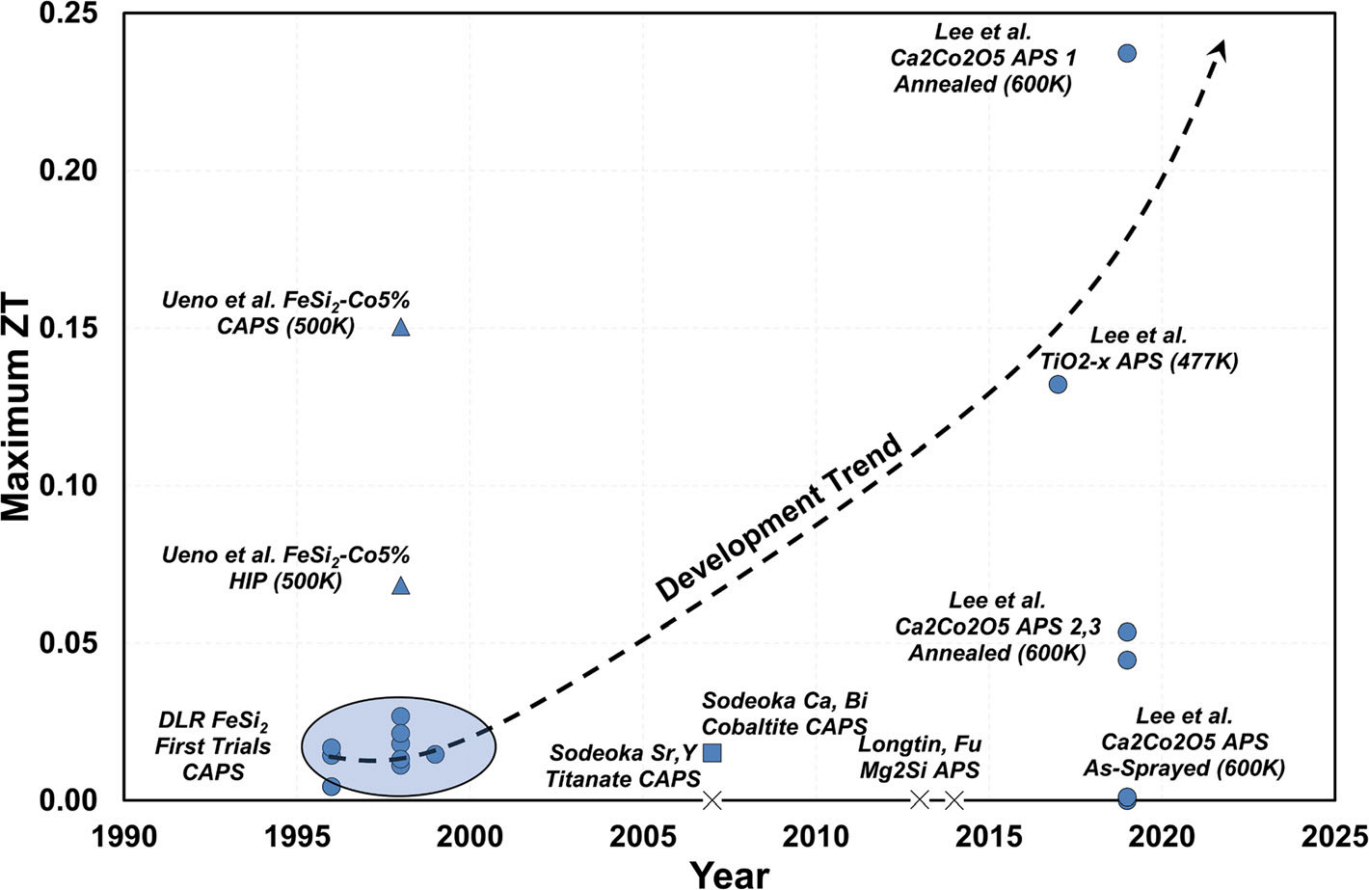
Maximum observed ZT of thermally sprayed thermoelectric devices published in the available literature vs. the year of publication. The overall trend suggests devices are continuously improving in their capabilities. The data were extracted from Ref 109, 113-121, 125, 126, 130, 133, 134

Clearly, the volume of literature in this field of thermally sprayed thermoelectric materials is still growing and can be considered to be maturing. However, the results to date are promising in showing that (1) thermal spray processing is a viable pathway for thermoelectric materials to yield competitive $ZT$ values and (2) the versatility and flexibility of the thermal spray process allow one to integrate p- and n-type thermoelectric materials together to spray-form a generation device entirely by one process. The general trends in development of thermally sprayed thermoelectric materials are captured in Fig. 9 and Table 1, wherein the relevant thermoelectric properties were extracted from the overviewed literature and the maximum available $ZT$ values were also calculated from the extracted properties. As shown in the Figure, in two decades, the $ZT$ values have seen marked improvement from initial studies with plasma-sprayed silicides. Nevertheless, for continued improvement, there is a need for innovation and breakthrough technology to enable the next growth in $ZT$ values.

**Table 1:** Extracted thermoelectric parameters from the available public literature for thermally sprayed thermoelectric materials. The data were extracted from Ref 109, 113-121, 125, 126, 130, 133, 134

*T*, C	Year	Material and author published	Seebeck coefficient, µV/K	Resistivity, 10^−3^ Ω cm	Thermal conductivity, W/m K	*ZT*
27	1996	Schilz et al. FeSi_2_ APS As-sprayed	180	4.55E + 01	1.5	1.43E-02
27	1996	Schilz et al. FeSi_2_ APS heat treated	180	5.00E + 01	4.5	4.32E-03
27	1996	Schilz et al. FeSi_2_ HIP	165	1.00E + 01	4.9	1.67E-02
27	1998	Schilz et al. FeSi_2_-Co_2_% CAPS	275	4.55E + 01	4.5	1.11E-02
27	1998	Schilz et al. FeSi_2_-Co_5_% CAPS	195	1.82E + 01	3.5	1.79E-02
27	1998	Tsutsumi et al. FeSi_2_-Co_2_% HIP	245	1.72E + 01	8.0	1.31E-02
27	1998	Ueno et al. FeSi_2_-Co_5_% CAPS	200	1.00E + 01	4.5	2.67E-02
27	1998	Ueno et al. FeSi_2_-Co_5_% HIP	165	8.00E + 00	4.8	2.13E-02
500	1998	Ueno et al. FeSi_2_-Co_5_% CAPS	215	6.25E + 00	3.8	1.50E-01
500	1998	Ueno et al. FeSi_2_-Co_5_% HIP	180	6.67E + 00	5.5	6.83E-02
27	1999	Huang FeSi_2_-Co_5_% HVOF JP5000	180	1.67E + 01	4.0	1.46E-02
27	2007	Sodeoka et al. Ca,Bi Cobaltite CAPS	150	2.40E + 01	1.9	1.50E-02
27	2007	Sodeoka et al. Sr,Y Titanate CAPS	155	8.00E + 03	11.0	1.00E-05
27	2013	Longtin et al. MgSi_2_ APS 01	55	1.11E + 04	3.8	2.15E-06
27	2013	Longtin et al. MgSi_2_ APS 02	78	1.43E + 04	4.0	3.19E-06
27	2014	Fu et al. Mg_2_Si APS 01	20	9.09E + 01	3.8	3.47E-05
27	2013	Fu et al. Mg_2_Si APS 02	550	1.67E + 04	2.0	2.72E-04
477	2017	H. Lee et al. TiO_2_-x APS	230	1.82E + 01	1.7	1.32E-01
600	2019	H. Lee et al. Ca_2_Co_2_O_5_ APS 1	5	2.00E + 04	2.1	5.20E-08
600	2019	H. Lee et al. Ca_2_Co_2_O_5_ APS 2	5	2.00E + 04	2.1	5.20E-08
600	2019	H. Lee et al. Ca_2_Co_2_O_5_ APS 3	700	2.00E + 04	2.1	1.02E-03
600	2019	H. Lee et al. Ca_2_Co_2_O_5_ APS 1—annealed	195	1.00E + 01	1.4	2.37E-01
600	2019	H. Lee et al. Ca_2_Co_2_O_5_ APS 2—annealed	175	2.08E + 01	2.4	5.35E-02
600	2019	H. Lee et al. Ca_2_Co_2_O_5_ APS 3—annealed	175	2.50E + 01	2.4	4.46E-02

One of the most limiting steps in advancing thermally sprayed thermoelectric research is the availability of quality feedstock material. For instance, powder feedstocks for thermally sprayed thermoelectric materials are still to this day primarily being synthesized in-house by researchers and laboratories. This results in powder morphologies and size distributions, which are typically not suitable for the thermal spray processes. One could consider if powder manufacturers were to collaborate with the researchers the quality of coatings research and subsequently the available $ZT$ values of spray-formed thermoelectrics could advance even further.

## Applications in Gas Sensing and Separation

The previous sections of this overview have shown how the utility and versatility of thermal spray technology can provide coating solutions in specific industrial applications. In this section, a broad overview will be given that demonstrates how thermal spray technology can facilitate the growth of clean and environmentally friendly industrial technologies. Specifically, gas sensing for emission monitoring and passive gas separation membranes and how thermal spray technology has played a role in their development will be discussed.

### Thermal Spray Coatings for Gas Sensors

The emission of harmful by-products from automobiles and industrial factories such as nitrogen oxides and carbon monoxides poses one of the most pervasive threats to the planet’s environmental stability (Ref 136-138). Emissions of these gases contribute to the production of acid rain, smog, and the erosion of the ozone layer in the atmosphere (Ref 136-138). As such, measures have been taken in countries across the globe to limit, mitigate, and reduce overall emissions of these harmful species (Ref 139-142). However, in order to verify these restrictions are met with some degree of accuracy, quantitative measurement of the emissions is necessary. For this, selective gas sensors are the technology of choice to quantify and identify when/if emission of harmful gases is detected. However, the accuracy and precision of commercially available gas sensors can be highly variable, and most state-of-the-art sensors require an integrated heater to optimize the material’s sensitivity to gas adsorption. These technological limitations are what fuel the continued search for improved materials as well as materials processing solutions—to further enhance the capabilities and operational capacities of the technology. Here, thermal spray technology and its role in gas sensor research will be overviewed to demonstrate the utility and opportunity of industrial-scale adoption.

#### Early History in Thermally Sprayed Gas Sensors

One of the first mentions of using thermal spray technology in the context of gas sensing is credited to Sunano et al. in 1985 (Ref 143). This US Patent illustrates the use of thermally sprayed titanium oxide deposited by plasma spraying onto the tip of two electrodes. In this case, the titanium oxide-sprayed coating acts as the sensor and responds to the presence of oxygen. Interestingly, the semiconducting properties of TiO_2_ and the decomposition to TiO_2-x_ by plasma spraying that are discussed in the thermoelectric Sect. "Applications in Waste Heat Reclamation and Energy Production" are again listed in this patent as the primary mechanism by which sprayed TiO_2_ can sense gaseous species. According to Sunano et al., the resistance of the plasma-sprayed TiO_2_ coating consistently increases with the presence of oxygen (Ref 143). In addition, Sunano et al. explain that conventionally available gas sensors (i.e., those formed by screen printing or sintering methods) have response time issues due to the contamination of organic solvents derived from the processing route (Ref 143), whereas plasma-sprayed TiO_2_ sensors possess two distinct advantages: they are inherently porous (ideal for gas adsorption and sensing), and they can be made without organic solvent contamination. In this patent, Sunano et al. further go on to explain that by heat treating the TiO_2-x_ coating in oxygen atmosphere at elevated temperatures, pure TiO_2_ can be recovered and the sensor response time is then more comparable to other available devices.

This initial example by Sunano et al. shows how the general versatility and capabilities of the thermal spray processes allow it to be considered for applicability in new markets. Madou et al. of Teknekron Sensor Development Corporation in California in 1992 also demonstrated the feasibility of plasma spray technology by showing the response of plasma-sprayed LaF_3_ in the presence of oxygen (Ref 144). The group goes further to state that they envision the successful production of multilayer solid-state ionic devices (i.e., for a humidity sensor) by this processing technology. In this case, the group envisioned spray-forming layers of electrical conductors, insulators, and the LaF_3_ sensing element into one package to accurately detect oxygen; however, it was never proven by this group in the subsequent literature.

#### SnO_2_-Based Gas Sensors by Thermal Spraying for Harmful Emissions Detection

The field of gas sensing technology is as diverse as the myriad of different gaseous species that are of interest to detect. While the prior examples demonstrated sensitivity to gaseous oxygen, as previously mentioned, there has been significant interest over the last several decades to reduce the emission of harmful gaseous species such as NO_x_ and CO. Shi et al. from Japan in 2003 allude to the Japanese government’s strict limitation on 40-60 parts per billion (ppb) emission of NO_2_, and the need to quantitatively detect these emissions (Ref 145). In this work, the group focuses on radio frequency (RF)-induced plasma spraying to deposit SnO_2_ sensing elements on alumina substrates. Semiconducting SnO_2_, specifically the rutile structure cassiterite, has been found to have environmentally dependent surface chemistries, be stable at high temperatures, and act as an effective sensor in the presence of various gaseous species (Ref 146). The work by Shi et al. mixed palladium with SnO_2_ powder and then deposited the coatings with RF plasma deposition at various conditions to find that the sprayed sensor was highly sensitive toward NO_2_ down to 20 ppb concentration (Ref 145). The high sensitivity at low gaseous concentrations was attributed to the uniquely fine grain size achieved with rapid solidification deposition.

Another example of thermally sprayed SnO_2_ gas sensors comes in 2007 from Chien et al. (Ref 147). Here, solution precursor plasma spray (SPPS) is utilized as the fabrication process. In SPPS, there are advantages in the ability to deposit nanostructured feedstocks that can form nanostructured coatings (Ref 147). Furthermore, SPPS has been proven in the past to be used for synthesis of unique coating chemistries that are otherwise unfeasible by conventional plasma spray deposition methods. In the study by Chien, a Taguchi Design of Experiment was utilized to screen the various spray processing parameters to find an optimal SnO_2_ spraying condition (Ref 147). Here, the sensitivity to ethanol vapors were studied. It was found that the SPPS process was capable of producing nanoporous structures, which contributed to the coatings’ high sensitivity to ethanol (Ref 147).

Iizuka et al. also used RF plasma technology to deposit SnO_2_-sensing thick films in order to detect volatile organic compounds (VOCs) that form during construction of new buildings (Ref 148). In this case, the authors introduced additional oxygen gas in the RF vacuum chamber to deposit coatings at a constant total pressure of 26 kPa. It was found in this study that by introducing additional oxygen to the process, the microstructure of the SnO_2_ coating could change from granular to a more uniform columnar structure. Furthermore, this study revealed that the deposition process used here could deposit sensing films, which are more sensitive and more effective at detecting low ppm concentrations than the alternative sol–gel produced films (Ref 148). The group followed up on this work one year later to find that there is a film/coating thickness effect—with lower thickness, the responsiveness increases (Ref 149). Furthermore, the group also was able to model the sensor response and in turn optimize the geometry and microstructure of the sensor (Ref 149). Finally, in 2013 the same group showed that by infiltrating the spray-formed SnO_2_ microstructure with 1 mol.% platinum, the minimum temperature for sensing reduced and the overall minimum sensing concentration capabilities increased from 40 to 20 ppb (Ref 150).

Spray-formed SnO_2_ gas sensors would then be seen again in the public literature around 2018 when Ambardekar et al. studied APS SnO_2_ coatings for sensing and catalyzing toxic gases (Ref [151]). Here, rather than using RF plasma deposition methods, Ambardekar uses traditional APS with a nitrogen–hydrogen plasma to melt and deposit SnO_2_ powder particles on alumina substrates. In this work, it was found that the nitrogen-only plasma was a more suitable spraying condition as opposed to incorporating secondary hydrogen gas. The authors point this effect to be due to morphological changes, but it is also possible that the incorporation of hydrogen causes stoichiometric and crystalline decomposition, which would inhibit the sensing abilities of the oxide coating (Ref 151). Nevertheless, the authors still show reasonable sensing capabilities for ethanol between 25 and 300 ppm; however, in this study, the SnO_2_ coating is cross-sensitive to other vapors as well (i.e., acetone), limiting its applicability in an industrial system.

Interestingly, the cross-sensitivity of plasma-sprayed SnO_2_ sensor coatings proved to be beneficial in 2019 when a study again by Ambardekar et al. showed SnO_2_ fabricated by plasma spraying to be sensitive to multiple diverse gases besides the hydrocarbons mentioned previously (Ref 14). In this study, SnO_2_ is again formed by plasma spray deposition, but the coatings are evaluated for their ability to sense hydrogen gas. It was found that the cross-sensitivity of the SnO_2_ coating to sense other gaseous species (i.e., CO and CH_4_) was temperature-dependent (Ref 14). This behavior indicates that either plasma-sprayed SnO_2_ can be tuned to be either multifunctional to sense multiple gases or selectively detect only hydrogen gas. Nevertheless, the benefit of rapid deposition by plasma spraying is noted and the scalability of such a system to an industrial scale is mentioned in this study (Ref 14).

#### Alternative Thermally Sprayed Gas Sensors Beyond SnO_2_

While SnO_2_ is a highly attractive candidate for gas sensing functionality, there have been additional studies that consider alternative gas-sensing semiconductors for fabrication by thermal spray to function as gas sensors. For instance, before thermal spray deposition was considered, one of the earliest materials proposed to have gas sensing capabilities was zinc oxide (ZnO) (Ref 152). Seiyama et al. in 1962 published in *Analytical Chemistry* the rapid change in electrical conductivity of thin ZnO semiconducting films in the presence of gaseous species at elevated temperatures. One of the primary limitations from widescale adoption of ZnO gas sensors was the relatively high working temperature required for optimal performance (~ 400-500 °C) (Ref 152). Equally limiting is the need for ZnO sensors to be selective to specific gases (depending on the application needs).

Thus, there is an active field of research dedicated toward improving these ZnO-based sensors in these regards to enable industrial adoption. Based on the enhanced gas sensing performance of screen-printed ZnO and ZnO coatings formed by pulsed laser deposition, Zhang et al. implemented APS to deposit ZnO coatings on an instrumented alumina substrate to study their gas sensing capabilities (Ref 153-155). These results showed that the optimal working temperature of the sprayed ZnO sensor was beneficially reduced to around 300 °C and improved sensor response in moist atmospheres was observed (Ref 153). Furthermore, the selectivity of the ZnO sensor to NO_2_ to H_2_ and CO gas was acceptable.

Further improving on these results, Zhang et al. went on in 2015 to study the formation of ZnO gas sensors by SPPS as opposed to APS (Ref 156). According to the authors, the flexibility and unique microstructures that SPPS can provide were driving forces toward switching the processing technology (Ref 156). Here, in this study, nanostructured and well-crystallized hexagonal ZnO were obtained by SPPS. Furthermore, the operational temperature of the ZnO sensor was further reduced from ~ 300 °C in the previous study to now ~ 250-325 °C; the ZnO SPPS coating was not as sensitive to the relative humidity of the surrounding environment as in previous studies (Ref 156). Also of note, the SPPS-processed coating showed higher selectivity to NO_2_ and was not influenced by the presence of other gases (i.e., SO_2_, NH_3_, CO, H_2_). In 2016, to push the capabilities of the system further, Geng et al. used SPPS with CdS to form CdS-ZnO coatings that were then post-treated in H_2_O_2_ to form ZnO_2_, which was then annealed at 400 °C to eliminate the ZnO_2_. The net result was confirmed by the authors to create oxygen vacancies and form CdS-ZnO_1-x_ (Ref 157). The result was a further improved response and recovery time of the modified sensor coatings as compared to the previous results.

One of the driving forces for the limitations of widescale adoption of ZnO as a suitable gas sensor material is the inherently high working temperatures to adequately sense impinging gases (Ref 158-160). It has been suggested that rather than using heater circuitry, that incident light can be used as an energy source to facilitate the adsorption and reaction of the semiconductor surface with incident gases (Ref 161). However, due to the wide band gap of stoichiometric ZnO, high-energy UV illumination would be required to photo-catalytically activate the surface. Therefore, in 2017 Zhang et al. proposed the use of SPPS to produce sub-stoichiometric ZnO_1-x_ directly—which would then be more susceptible to surface heating by incident visible light (Ref 158). The results from this study suggested the hypothesis set forth by Zhang was accurate—the ZnO_1-x_ coatings were overly sensitive to incident NO_2_ gas with added photon impingement. And, furthermore, the formation of oxygen vacancies appeared to be a leading driver for NO_2_ adsorption at the vacancy sites (Ref 158). Finally, this method was further refined in 2020 and Zhang et al. showed that SPPS ZnO_1-x_ coatings could sense NO_2_ at the ppb level under blue light illumination at room temperature—essentially eliminating the need for auxiliary heating circuitry (Ref 162). This example of the progression of gas sensing coating technology shows how through processing refinements, new and emerging markets can become viable opportunities for thermal spray.

Other noteworthy achievements in the field of alternative thermally sprayed gas sensors include work done in 2013 by Gardon et al. in Spain—wherein they successfully deposited a sub-stoichiometric TiO_2-x_ coating by APS (similar to what was described in the 1985 patent by Sunano et al.) onto a flexible polymeric substrate without degradation of the polymer (Ref 163). Furthermore, the flexible sensor was able to detect NH_3_ gas ranging from 10 to 100 ppm due to the surface oxygen vacancies and intrinsic coating porosity (Ref 163). In addition, the group of Zhang et al. also studied SPPS tungsten oxide (WO) gas sensing coatings using similar approaches to what was done for ZnO gas sensing coatings (Ref 164).

Evidently, the field of gas sensing coatings has a rich and diverse amount of literature currently available to the public. However, the technology can still be considered to be maturing. While marked improvements in coating performance have been catalogued, these systems have still not infiltrated the industrial sector.

The lack of industrial adoption may imply that there are still improvements to be made in coating processing and design. One possibility would be to consider fabricating the entire gas sensor by thermal spray—including the insulating substrate. By utilizing cheaper substrate materials (i.e., aluminums, steels, etc.) and depositing the insulating layer (i.e., alumina/spinel) prior to the gas sensor, cost benefits can be envisioned. One can also consider depositing the heating circuitry as well as the gas sensor and insulator by thermal spraying. As a result, the entire gas sensor system would be fabricated using one process, inevitably reducing production costs and complexities, which may assist in the industrial adoption of thermal spray in this market.

### Thermal Spray Coatings for Gas Separation

Another field that is emerging and related in application scope is the development of membranes for the selective separation of gaseous species. The concept behind gas separation membranes derives from the idea of separation of gaseous species by different adsorption mechanisms. Generally speaking, there are three predominant ways in which gaseous species can be separated while passing through a membrane (Ref 165). They are:

- Knudsen diffusion—wherein separation is based on the inverse square root ratio of the molecular weights of two gaseous species. For Knudsen diffusion to occur, the pore diameter must be at least ten times smaller than the mean free path (Ref 166).
- Microporous molecular sieving—wherein separation is based on the much higher diffusion rates of the smallest molecule. Is not highly effective with similarly-sized species (i.e., N_2_, O_2_)
- Solution-Diffusion Separation—wherein separation is based on both the solubility and mobility factors. Diffusivity selectively favors the smallest molecule; solubility selectively favors the most condensable molecule

In the field of gas separation membranes, the most well-known membranes to date are those that were developed in the 1970s and 1980s by the Monsanto Corporation in St. Louis, Mo (Ref 167, 168). These membrane systems were able to separate hydrogen from the nitrogen, argon, and methane in the purge gas of ammonia synthesis plants. However, these membranes, and the ones that followed from Monsanto (which were able to separate nitrogen from air, CO_2_ from natural gas, and hydrogen from various refinery and steam by-products) would be comprised of polymeric materials and are thus restricted to lower operating temperatures. These commercially accepted devices are all dense membranes that operate based on the solution-diffusion mechanism listed above, wherein the gas enters the membrane and penetrates as a liquid, and then filtration occurs through-thickness as the gas passes through the membrane by means of a concentration gradient (Ref 167).

While these polymeric solution–diffusion membranes are well-established and accepted by the industry, there are a number of industrial processes wherein polymeric materials are not acceptable, yet the use of a gas separation membrane would still be beneficial. Specifically, processes that take place in extreme environments (i.e., chemical reactors, combustion processes, etc.) are not suitable locations to implement polymeric membrane systems yet process efficiency and environmental impact might benefit from separation membrane implementation. There is, as a result of this need, a driving force to develop and establish so-called *inorganic membranes*. Examples of these types of membranes include dense metallic membranes (i.e., palladium, palladium alloys, silver, etc.), which are selectively permeable to only one kind of gaseous species—such as hydrogen or oxygen (Ref 169). Alternatively, semiporous membranes are also considered. These would incorporate the use of ceramic materials (i.e., alumina, silica, titania, glass, etc.) and are characterized by their high permeability, but low selectivity. When considering the structure of a gas separation membrane, typically there is a support (i.e., substrate), followed by the separation membrane (Ref 170). Typical processing methods for these separation membranes, specifically the ceramic variants, include bulk synthesis methods such as sintering, tape casting, etc. However, these processes are met with inherent disadvantages—in that the assemblage of highly dense ceramic membranes on metallic supports can elicit thermal expansion-induced macrocracks that render the separation membrane effectively useless (Ref 170-172). One can consider based on the materials and microstructures of interest for this application that thermal spray technology can contribute to the growth and evolution of gas separation membranes. However, there has only been limited published work on using thermal spray technology to fabricate gas separation membranes.

To the authors’ knowledge, only one group of researchers specifically devoted studies toward fabricating and developing a gas separation membrane by thermal spray technology. In 2009, Mauer et al. published an article in the *Journal of Thermal Spray Technology* demonstrating a new processing capability—very low pressure plasma spraying (VLPPS) (Ref 173). In this methodology, the plasma jet emitting from the torch has very weak and limited interaction with the surrounding environment. This unique feature of the VLPPS process enables thermal spraying of materials that would otherwise not survive the intense melting/solidification process of other conventional plasma spray methods.

The ability to deposit unique materials with VLPPS presented an opportunity to demonstrate a sprayed gas sensor. In particular, perovskite materials are interesting in the context of gas separation for their high mixed electronic and ionic conductivity (MEIC) (Ref 173). In gas separation membranes, mixed ionic and electronic conductors have been conceptualized for decades. This mechanism has been observed by Nernst in 1899 for Y_2_O_3_-ZrO_2_ solid solutions (Ref 174), and the engineering application of the principle has been refined over the years (Ref 175-177). One of the most presently popular implementations of this principle is in the industrial solid oxide fuel cells (SOFCs) and industrial-scale membrane reactors (Ref 176-180). However, generally speaking, perovskite materials when made into feedstock powders for thermal spraying will decompose easily when melted by the plasma jet. Consequentially, plasma-sprayed perovskites are often sub-stoichiometric, similar to what is observed in plasma-sprayed TiO_2-x_ and ZnO_1-x_ as described in the prior section(s). One can consider utilizing mild spray conditions to retain the stoichiometry, yet at the same time, oxygen-ion-conducting membranes such as these must be highly dense to be efficient, which inherently requires high-plasma enthalpy conditions and complete melting of the particles.

In the work presented by Mauer in 2010, x-ray diffraction analysis revealed that even the VLPPS coatings comprised of perovskite Ba_0.5_Sr_0.5_Fe_0.2_Co_0.8_O_3_ were highly sensitive to decomposition despite the vacuum deposition environment. As stated in the study, there is a fine line between full melting and deposition of dense coatings and phase decomposition due to the spray process (Ref 173). From this study, the most influential parameter on the decomposition of the material was the plasma gas chemistry. Despite not using hydrogen, decomposition of the perovskite material was still observed, and the gas-tightness of the coating was not optimized (Ref 173).

In 2013, Jarilgo followed up on this initial work and again studied the feasibility of plasma-spraying perovskite materials for oxygen transport membranes (Ref 181). In this case, La_1-x_Sr_x_Co_y_Fe_1-y_O_3-δ_ (LSCF) was the chosen perovskite material for its good thermal stability in conjunction with high MEIC (Ref 182). In this case, plasma spraying under very low pressures was again utilized (Ref 181). Unlike the prior study by Mauer, the coatings achieved here were highly dense, gas-tight coatings (Ref 181). However, x-ray diffraction analysis revealed the formation of metastable and dissociated phases. Nevertheless, in this case, annealing the coatings allowed for the removal of some of these metastable phases and furthermore increased the crystallinity of the perovskite phase (Ref 181). The result was a plasma-sprayed membrane on a porous support, which was able to induce an oxygen flux comparable to a reference membrane (Ref 181). As such, this study serves as one of the first to validate the use of thermal spray technology as a simplified, low-cost alternative to more conventional synthesis methods (i.e., screen printing, tape casting, etc.), which require more post-fabrication steps and delicate control of the layers.

**Fig. 10:**
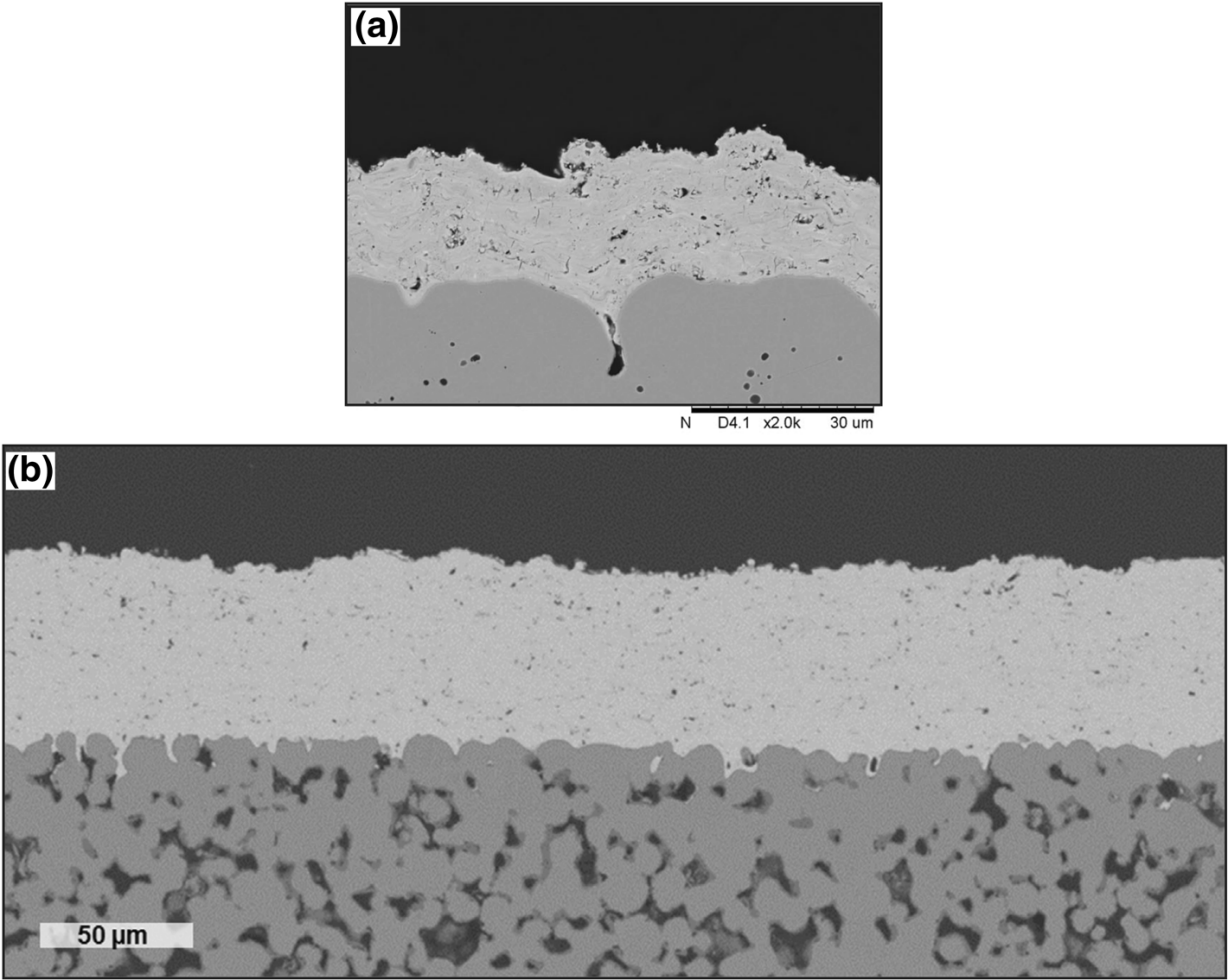
Thermally sprayed gas separation membranes. (a) Suspension plasma-sprayed LCSF on a porous metallic substrate support (Ref 183). Reprinted with permission from E.S.C. Fan, O. Kesler, Deposition of Lanthanum Strontium Cobalt Ferrite (LSCF) Using Suspension Plasma Spraying for Oxygen Transport Membrane Applications, Journal of Thermal Spray Technology, Volume 24, pp. 1081-1092, 2015, Springer Nature. (b) PSPVD LCSF separation membrane deposited on porous Crofer steel substrate (Ref 184). Reprinted from Separation and Purification Technology, Volume 219, M.E. Ivanova, W. Deibert, D. Marcano, S. Escolástico, G.Mauer, W.A. Meulenberg, M. Bram, J.M. Serra, R. Vaßen, O.Guillon, Lanthanum tungstate membranes for H_2_ extraction and CO_2_ utilization: Fabrication strategies based on sequential tape casting and plasma-spray physical vapor deposition, pp. 100-112, Copyright 2019, with permission from Elsevier

Following the initial work by Jarilgo et al., there have been some subsequent studies to examine the feasibility of thermal spray technology to synthesize oxygen-conductive membranes. In 2015, Fan et al. studied the fabrication of LSCF oxygen membranes using suspension plasma spray (SPS) techniques (Ref 183). Unlike Jarilgo et al.’s study, SPS is a more traditional thermal spray deposition technology in the sense that coatings are formed by the stacking and rapid solidification of molten droplets. Furthermore, Fan et al. found that under certain spraying conditions (i.e., short standoff distances) the LCSF coatings undergo through-thickness vertical cracking due to the thermal stresses (Ref 183), while, on the other hand, longer standoff distances yielded smooth, dense coatings. In this study, there was an issue as a consequence of the porous substrate geometry—wherein the SPS deposit could not easily form without inducing pinhole-type defects in the coating. This was mitigated by optimizing the total thickness of the coating. Ultimately, it was again found that the SPS-fabricated LSCF oxygen transport membranes performed competitively to reference materials (Ref 183). It is important to note these SPS-fabricated membranes were not explicitly measured for their oxygen permeability. Cross-sectional images of some of these membranes are shown in Fig. 10. In showing the cross-sections of these two unique deposits, it is important to recognize that despite the fact that there is still some degree of micro-porosity in the thermally sprayed deposits, the overall performance of the gas separation membranes made by this process was comparable to commercially available systems (Ref 183).

These initial studies demonstrated the feasibility of using thermal spray technology to fabricate oxygen transport membranes and can be considered as framework studies to enable optimization and potentially industrial adoption of thermally sprayed membranes in the future. Recently, in 2019 Ivanova et al. published work on direct comparisons between plasma-sprayed and (more conventionally) tape-casted/screen-printed gas separation membranes (Ref 184). In this work, lanthanum tungstate LAWO membranes were fabricated for H_2_ extraction and CO_2_ utilization. Here again, thermal spray processes in low pressure was chosen to fabricate the layers. It was demonstrated that gas-tight 25-60 µm-thick LAWO membranes could be achieved (Ref 184). The results showed high phase-purity in the spray-fabricated membrane and additionally the H_2_ flux through the system was comparable for both the tape-cast system and the sprayed system. Also in this study, Ivanova et al. pointed toward long-term durability issues such as delamination of the sprayed layer and improved phase-stability during deposition (Ref 184).

Later, a study by Marcano et al. would demonstrate modified experiments wherein the spraying parameters were further tuned and the effect of the addition of O_2_ in the chamber would be studied (Ref 185). The results from this study suggested that the addition of O_2_ allowed the formed coating to have a more relaxed lattice constant, which indicated more phase-pure cubic LAWO. Furthermore, it was determined that colder processing conditions overall promoted the formation of the correct phase during deposition, and the resulting gas-tightness of the coatings was highly competitive to commercial membranes. Clearly from these preliminary works, there is indeed a possibility for thermal spray technology to be adopted in gas separation membranes.

This section has shown that thermal spray technology has potentially useful versatility and utility in the applications of both gas sensing and gas separation. It can be seen that as the research matured, these coating systems were continuously optimized for their performance and functionality. However, despite these performance gains, thermally sprayed gas sensors and separation membranes still have not seen adoption in the industry. One can argue, taking insights from the polymer community and a recent review in 2020 by Corrado et al., that the field of gas separation membranes in particular is still limited in commercialization by two factors: the tradeoff between permeability and selectivity and the long-term diminished performance caused by physical aging (Ref 186). Nevertheless, the results presented here show a high level of promise for industrial gains, should these thermal spray gas separation membranes be adopted.

## Applications in Extreme Environments

At the surface, the concept of ‘thermal spray coatings for extreme environments’ can be somewhat ambiguous, given the vast array of existing applications wherein the working environment is highly aggressive (i.e., paper rolls, hydraulics, sliding wear, etc.). In this section, the primary focus will be to highlight two key ‘extreme environment’ applications, which are both evolving, and research in thermal spray coatings for these fields has a rich historical background. In doing so, the purpose is to illustrate two established coating applications, which have successfully undergone technological evolutions that overcame the risks of industrial adoption in an already high-risk environment. These two exemplar case studies are coatings for ultrahigh temperature, hydrogen-firing turbines and ultrahigh temperature reentry systems.

### Thermal Spray Materials for Ultrahigh Temperature and H_2_ Turbines

The concept of thermally sprayed coatings for high temperature protection in gas turbine engine components is not new on its own. Decades of research have gone toward understanding the process–property–performance relationships behind thermally sprayed thermal barrier coatings (TBCs) (Ref 17-19, 30, 31, 187-196). Thanks to these efforts, key understandings in the durability mechanisms and benefits of materials such as 7 wt.% yttria-stabilized zirconia (7YSZ) have been established. For instance, one of the foundation-establishing works by Stecura, which demonstrated the markedly high-performance of plasma-sprayed 7YSZ as compared to other YSZ oxides of varying yttria content (Ref 17). More recently, works such as those by Krogstad et al. built on these original findings and showed the high-temperature phase stability of 7YSZ and elegantly captured its decomposition mechanisms due to high temperature exposure, which allowed manufacturers to understand and therefore predict/design coatings around these expected degradation mechanisms (Ref 197).

However, as turbine temperatures continue to increase, the feasibility and applicability of traditional plasma-sprayed 7YSZ coatings continue to diminish. This implies a significant need for either materials or coating innovation to enable turbine temperatures to continue to increase for the sake of efficiency, power output, and reduced emissions. From the materials side, there is interest in integrating low-thermal-conductivity oxides as new thermal barriers (i.e., gadolinium zirconate, GZO) (Ref 198-201). However, integrating these new materials as thermal barriers has proven in the past to be somewhat challenging. As one example of a design challenge, GZO has been observed to be highly incompatible with the aluminum oxide thermally grown oxide (TGO) scale that forms on most MCrAlY bond coats in TBC systems (Ref 202-204). Work has been shown in the past by Vassen et al. in the late 2000s, Viswanathan et al. in the mid-2000s, and others, where integrating 7YSZ in tandem with GZO into a single coating system can overcome these issues, providing performance benefits while also allowing the implementation and integration of new TBC oxides (Ref 29, 31, 190, 191, 205-209).

These continued advancements in TBC technology have allowed current state-of-the-art natural gas firing gas turbine engines to continue evolving in order to operate globally as a source of power for various nations. However, there is a continued push to improve the efficiency as well as reduce emissions from these technologies. This most recent push for further improved turbines comes from delays in the advent of renewable clean energy sources as the primary energy supplier in the world (Ref 210-214). One way to achieve improved turbine efficiency in conjunction with reduced emissions can be by implementing hydrogen-firing turbines in the power generation sector (Ref 215-217). The primary benefits of hydrogen turbines come from near-zero hydrocarbon by-products from operation. At the same time, manufacturers seek to increase the operating temperature, for the sake of further increased thermodynamic efficiency of the turbine engine.

It is therefore implied with high-temperature hydrogen turbines is that the operating temperatures, or the turbine inlet temperatures, will be in excess of 1800 °C (Ref 218-220). In such a case, traditional TBC-coated metallic superalloy components are no longer feasible—even with substrate film cooling technologies implemented. The thermally insulative capabilities of current state-of-the art TBCs are not sufficient enough to maintain the turbine component temperatures below threshold levels where creep relaxation deformation becomes a concern (Ref 221-223). Therefore, advances have become necessary both at the component level and the coating level.

Ceramic-based turbine components have been studied for decades, and the successful implementation of such components has only been realized in the last several years (Ref 224, 225). However, the ceramic components thus far are stationary and also not necessarily located at the hottest sections of the turbine. It is possible that future considerations must include the rotating and hot section components (i.e., turbine blades, turbine vanes, etc.). This implies that the coating requirements for the new substrate materials will also change.

One of the advantages of using ceramic components is the component cooling requirements subsequently change. CMCs are not as thermally limited as even the most advanced single crystal superalloy component and can consequentially withstand overall higher sustained temperatures by comparison (Ref 226, 227). However, the SiC–SiC CMCs currently used in today’s turbine engines are highly susceptible to volatilization in the presence of excessive water vapor, which is also the only by-product of a hydrogen combustion source (Ref 228-230). This therefore implies a surface coating is necessary to protect a component in a hydrogen turbine from steam oxidation and volatilization.

Protective coatings against steam oxidation have been studied heavily in the literature over the last several decades (Ref 231-233). Early work focused on aluminum silicates (i.e., mullite, BSAS), which had relatively good performance under water vapor environments (Ref 234, 235). However, unlike 7YSZ, these materials are relatively more brittle and subsequently are not as resistant to thermomechanical excursions. Most silicates have thermal expansion coefficients lower than 7YSZ, and furthermore, their thermal expansion coefficients are not as stable over as wide a temperature range (Ref 233, 236-239). However, for these preliminary works, the results were sufficient to prove there was efficacy in thermally spraying these materials for use in so-called environmental barrier coatings (EBCs).

The present state of research, however, has come to realize that these first-generation BSAS/Mullite EBCs are not sufficient candidates for long-term exposure in such environments (Ref 234, 240-242). Alternatively, rare-earth (RE) silicates such as ytterbium and yttrium silicates have been studied for their resistance to water vapor exposure (Ref 242-244). However, one challenge in the implementation of these materials as sprayed coatings is when plasma-spraying these materials by conventional means (i.e., APS), they form as amorphous coatings (Ref 245, 246). This requires a subsequent annealing/crystallization step that can induce cracking in the microstructure. Studies have shown that one way to avoid this is by spray-forming RE silicates as crystalline materials, which has been shown as accessible through different processing means than conventional air plasma spraying (Ref 245, 247-250).

In both the aforementioned cases of the first- and second-generation EBCs, most available studies of these materials in the literature do not consider the ultrahigh temperatures currently on the horizon. As an example, in most of these early studies, a silicon bond coat was always envisioned to be utilized for the silicon carbide CMC. However, silicon bond coats undergo severe softening and creep relaxation at temperatures around 1300 °C, posing a severe limitation in implementation at higher turbine firing temperatures (Ref 251-254). One mitigation strategy to avoid this temperature limitation is the implementation of oxide-based bond coats, which have been studied by NASA recently (Ref 251, 255, 256). Alternatively, one can consider the re-introducing of thermal barriers to the EBC in the form of multilayer structures for the sake of reducing the silicon bond coat temperature. For instance, low-conductivity oxides such as gadolinium zirconate can be envisioned as a constituent in the thermal barrier multilayer.

**Fig. 11:**
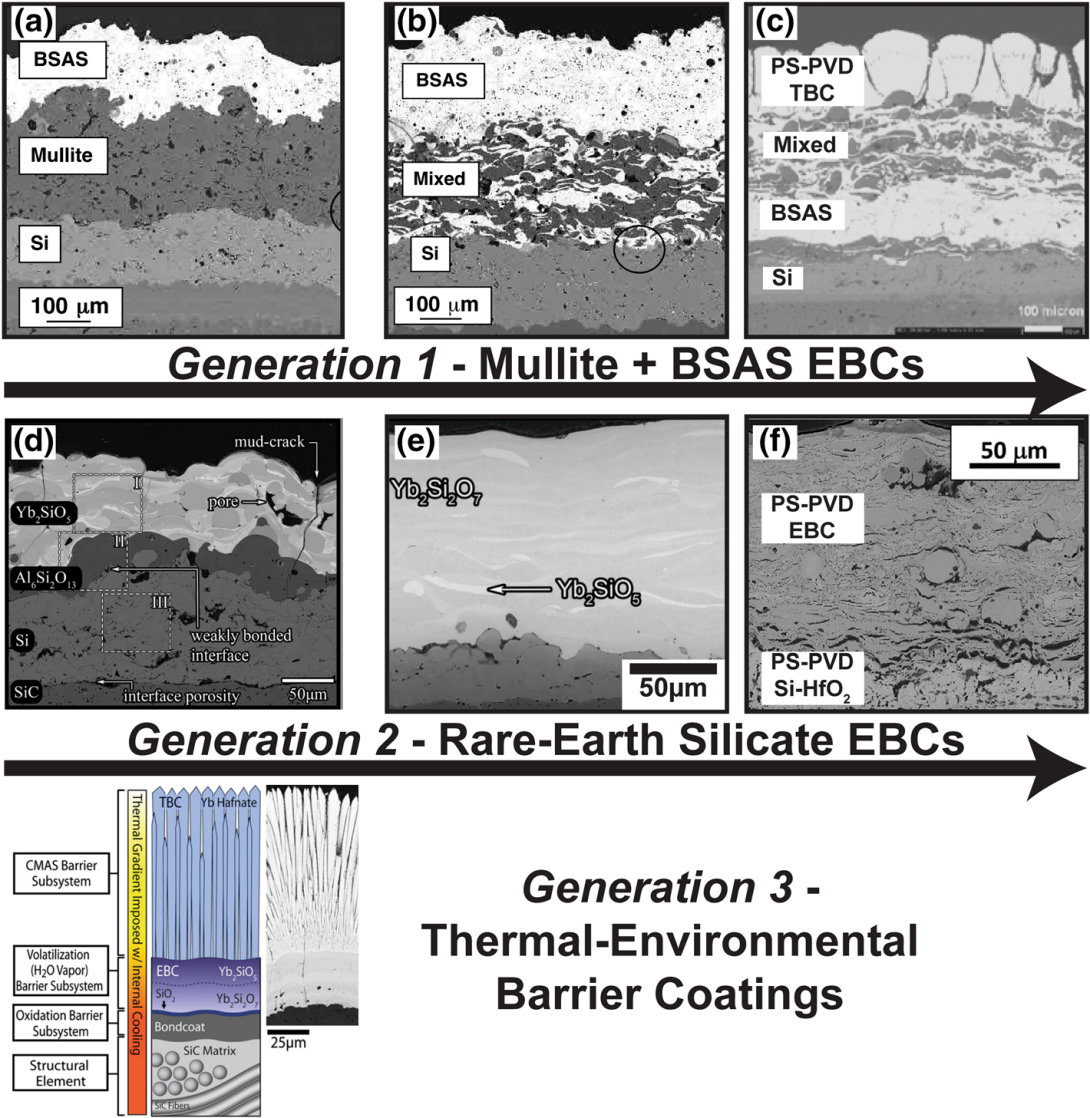
Progression of technology in thermally sprayed Thermal-Environmental Barrier Coatings. (a) Traditional BSAS-Mullite-Si EBC (b) Advanced BSAS + Mixed BSAS-Mullite + Si EBC (Ref 231). Used with permission of American Society of Mechanical Engineers (ASME), from Evaluating Environmental Barrier Coatings on Ceramic Matrix Composites After Engine and Laboratory Exposures, K.L. More, P.L. Tortorelli, L.R. Walker, J.B. Kimmel, N. Miriyala, J.R. Price, H.E. Eaton, E.Y. Sun, G.D. Linsey, Volume 4A, 2002; permission conveyed through Copyright Clearance Center, Inc. (c) First proposal of a thermal-environmental barrier coating, wherein a EBPVD TBC is deposited atop a similar structure to (b) (Ref 241). Used with permission of John Wiley & Sons—Books, from Thermal and Environmental Barrier Coatings for SiC/SiC CMCs in Aircraft Engine Applications*, I. Spitsberg, J. Steibel, International Journal of Applied Ceramic Technology, Volume 1, issue 4, 2004; permission conveyed through Copyright Clearance Center, Inc. (d) Ytterbium monosilicate EBC atop Mullite and a silicon bond coat (Ref 288). Reprinted from Journal of the European Ceramic Society, Volume 34, B.T. Richards, H.N.G. Wadley, Plasma spray deposition of tri-layer environmental barrier coatings, pp. 3069-3083, Copyright 2014, with permission from Elsevier. (e) Ytterbium disilicate EBC atop a silicon bond coat. The plasma-sprayed ytterbium disilicate coating is a heterogenous mixture of ytterbium disilicate and ytterbium monosilicate splats (Ref 289). Reprinted from Acta Materialia, Volume 106, Bradley T. Richards, Kelly A. Young, Foucault de Francqueville, Stephen Sehr, Matthew R. Begley, Haydn N.G. Wadley, Response of ytterbium disilicate–silicon environmental barrier coatings to thermal cycling in water vapor, pp. 1-14, Copyright 2016, with permission from Elsevier. (f) Advanced PSPVD EBC system which utilizes a modified Si-HfO_2_ bond coat for higher-temperature applications (Ref 242). The final structure of the envisioned Generation-3T-EBC is shown at the bottom. Reprinted from Journal of the American Ceramic Society, Volume 98, D.L. Poerschke, D.D. Hass, S. Eustis, G.G.E. Seward, J.S. Van Sluytman, C.G. Levi, Stability and CMAS Resistance of Ytterbium-Silicate/Hafnate EBCs/TBC for SiC Composites, pp. 278-286, Copyright 2015, with permission from Elsevier

The concept of a multilayer structure that protects a turbine component in a high-temperature, high-water–vapor environment such as in a hydrogen turbine has been termed a ‘thermal-environmental barrier coating’ (T-EBC) in the literature (Ref 7). T-EBCs have become of interest in the community for several reasons—including that the implementation of a thermal barrier layer within the coating structure would provide some form of thermal degradation resistance for the underlying substrate. Another attractive reason to consider T-EBCs is in the propulsion sector, where ingested foreign debris that form calcium-magnesium-alumino-silicates (CMAS) at high temperatures (such as those envisioned for future turbines) (Ref [257–261]). The integration of a large-cation oxide such as a thermal barrier oxide can promote long-term longevity of the T-EBC through chemical reactions with molten CMAS often found in propulsion systems (Ref 257, 259). Figure 11 pictographically shows this technological progression of thermally sprayed EBCs and T-EBCs as published in the literature over time.

According to Web of Science, since 2021, there has been as much as between 51 and 65 articles published in the literature on T-EBCs for next-generation turbine components (Ref 262). These articles are centered around: finding new candidate T-EBC materials, modeling the failure mechanisms under thermomechanical loading, and studying the efficacy of T-EBC materials under CMAS environments (Ref 256, 263, 264). However, there is limited research to this date on a (conventional) thermally sprayed multilayer T-EBC and its durability/response under highly simulative environments. As such, the continued growth and expansion of research in this field are expected to follow as hydrogen turbines proceed to become increasingly pervasive in the future.

### Thermally Sprayed Materials for Reentry and Hypersonics

As in T-EBCs, thermal spray high-temperature coatings have been considered for use in other environments that are also highly aggressive (i.e., high-temperature, high-pressure, etc.), but are not specifically in gas turbines. One of the earliest works published on thermally sprayed ‘extreme-environment coatings’ was in 1985 when Fournier et al. studied the transmission electron microscopy of plasma-sprayed titanium carbide (TiC) coatings (Ref 265). In the article, the authors cite this coating to be useful as it has a high melting point (~ 3340 K), and a low physical and chemical sputtering yield upon ion bombardment. In 1987, Dallaire et al. go on to say these plasma-sprayed TiC coatings are of interest among the nuclear community (Ref 266). Specifically, this is due to their ability to withstand high energy fluxes, as would be the case in fusion devices. In 1988, Brossa et al. go on to study the plasma-sprayed Al_2_O_3_, TiO_2_, TiC, and W-5%Re coatings, sprayed by vacuum plasma spraying, and ablated with an electron beam gun—again for applications in the walls of nuclear fusion reactors (Ref 267). From this work, the authors found that all the candidate materials have potential uses in a high-energy ablative environment, and go on to state that each coating can be used in a specific location/part of a nuclear reactor (Ref 267).

These early works on plasma-sprayed materials for extreme nuclear ablative environments can be considered as some of the initial works that set the framework for future studies for so-called ultrahigh temperature coatings (UHTCs) for hypersonics and re-entry. In the early 1990s, carbon/carbon composites were introduced as a feasible material for these applications, with original discovery of the materials dating back to the mid-1900s for use in American Defense systems. The carbon–carbon composites had interesting properties: low intrinsic density, high thermal conductivity, and excellent mechanical properties at elevated temperatures (~ 2000 °C) (Ref 268). However, these composites were limited in their application scope because they also possessed low hardness, high as-fabricated roughness, and low corrosion/erosion resistance which made them difficult to implement in ablative re-entry applications. In 1992, Boncoeur et al. studied the feasibility of applying a plasma-sprayed coating on carbon/carbon composites to improve their applicability in such extreme environments (Ref 269). In this case, HfC coatings deposited on carbon–carbon composites using CAPS were studied. It was determined using CAPS allowed the coating to retain the same structure and chemistry as the starting powder. The authors also determined the bonding strength between the coating and the carbon/carbon composite was so high that fracture during adhesion testing occurred in the substrate. Lastly, the authors also determined that the thermally sprayed coating showed improved hardness compared to the original carbon/carbon composite hardness, suggesting promise for thermal spray coatings in hypersonics.

Following this, in the *International Thermal Spray Conference* (ITSC) 2000, Valente et al. published a study on plasma-sprayed UHTCs for thermal protection systems (TPS) that would enable space vehicles to safely re-enter earth’s atmosphere. Considered here are diboride ceramics, which are known to have unique properties such as high-temperature stability, oxidation/thermal shock resistance, and good ablation resistance at 1700-2800 °C in the presence of high velocity dissociated air (Ref 270-272). This publication serves as one of the first studies on plasma-sprayed diborides. In this study, zirconium diboride ZrB_2_ particles with a silicon carbide SiC core were fabricated by spray drying and plasma-sprayed with CAPS. Here, a design-of-experiment (DOE) approach to study the thermal stability of plasma-sprayed diborides was taken. The first coating trials were subjected to thermal cycles by exposure to a plasma torch at 900 mbar pressure to a surface temperature of ~ 2000 °C for multiple cycles, and no fracture events were observed in the coatings.

The work by this group in Italy was further refined in 2002, where Bartuli et al. again study the ZrB_2_-SiC system sprayed by CAPS (Ref 273). In this work, detailed microstructural studies are done to ascertain the coating formation dynamics in an effort to understand the nature of splat buildup during deposition. According to the authors, the ZrB_2_-SiC is highly effective because the outer ZrB_2_ shell protects the inner SiC core during the plasma spraying, which allows for nearly complete retention of the volumetric content of SiC, which is otherwise impossible with thermal spray. Furthermore, under prolonged thermal exposure tests, it was found that the ZrB_2_-SiC composite material has a protective oxidation mechanism wherein zirconia, zircon, and eventually silica oxides form during thermal exposure (Ref 273). In response to these results, in 2003 researchers at the Italian Aerospace Research Center developed the first prototype conical carbon–carbon composite structure with a plasma-sprayed coating (Ref 274). That prototype nose cone incorporated a combination of plasma-sprayed ZrB_2_-SiC and sintered ZrB_2_-SiC layers and was durable enough to withstand thermal cycles in a simulative plasma jet rig. In 2008, the same structure was modeled by the group at CIRA Italy and the experimental results and modeling results were found to be in good agreement with each other (Ref 275).

From this point, the research on thermally sprayed diborides and other UHTCs for aviation and reentry becomes increasingly diversified and the volume of research itself also increases significantly. An impressive collection of the recent work conducted in thermally sprayed UHTCs has been published this year by Lynam et al. wherein a detailed overview of the spray methodologies, spraying conditions, and coating compositions is presented (Ref 276). What will be presented here is a brief overview of salient results, which capture the progression in technology, and additionally results which speak toward the opportunity in multilayer thermal spray engineering for re-entry and hypersonic coatings will also be discussed.

In 2012, Zou et al. illustrate the possibility of ZrB_2_-SiC and other like-diborides to be temperature-limited (Ref 277). In their study, it is hypothesized that hypersonic flight would elicit surface temperatures above 2273 K, where the diborides may not be as effective under long-term exposures. Of note here is this group studied coatings tailor-made for C/SiC composites, which are slightly different than the C/C composites studied earlier. Therefore, due to the proposed change in temperature requirements and change in substrate materials, it is suggested by the authors that alternate coating compositions must be considered. Interestingly, what is presented in this study is a double-layer thermally sprayed coating, comprised of ytterbium monosilicate (Yb_2_SiO_5_) at the substrate interface and lanthanum magnesium hexaluminate (LaMgAl_11_O_19_, LMA) at the surface layer. This configuration is designed to have good compatibility with the C/SiC substrate by utilizing ytterbium monosilicate for expansion mismatch mitigation as well as thermal barrier properties in using the LMA topcoat. Results from this study after representative thermal loading using an oxyacetylene torch suggested an 80% reduction in weight loss as compared to the uncoated components (Ref 277).

In 2013, Morks et al. propose another novel plasma-spray-formed multilayer structure, this time for traditional carbon–carbon composites, comprised of ZrB_2_ followed by mullite then yttria-stabilized zirconia (Ref 278). The rationale here is that the ZrB_2_-SiC composite diborides originally studied have poor fracture toughness due to the formation of the secondary phases. To mitigate this, the mullite interlayer in Morks et al.’s work is intended to soften, melt, and become viscous at ultrahigh temperatures. In doing so, the molten mullite can then infiltrate the ZrB_2_ and YSZ layers and improve the structure’s overall rigidity and toughness. While not experimentally proven in this published work, the proposal and demonstration of a multilayer design for improved functionality are still important in the context of this overview.

In some cases, for UHTCs, multiple processing routes are taken to fabricate a multilayer structure. This is most often desired when the researchers wish to incorporate an intermediary SiC layer, which is notoriously difficult to plasma spray due to the inability to melt and project SiC droplets onto substrates (Ref 274, 275). Liu et al. in 2014 combined a pack-cementation process SiC interlayer with a plasma-sprayed MoSi_2_ outer layer. Here, MoSi_2_ was chosen over ZrB_2_ because it is an equivalently strong candidate for anti-ablation coatings at ultrahigh temperatures (Ref 279). The results from this study suggested that MoSi_2_ forms protective SiO_2_ during simulative testing; however, it was found that this oxide scale can vaporize upon prolonged exposure at high temperature, and MoO_3_ can form in its place, which vaporizes at ultrahigh temperatures and can damage the coating structure (Ref 279).

Then, in 2017, Liu et al. publish again a study on anti-ablative coatings, this time utilizing the beneficial properties of ZrB_2_-SiC blended with MoSi_2_ (commonly referred to as ‘ZSM’ coatings) (Ref 280). However, in 2010, a study by Pulci et al. discovered that the ZSM coatings have a ‘softening’ point at around 1500 °C, which the authors postulated was due to combined sintering and self-healing effects at the elevated temperatures (Ref 281). Nevertheless, Liu et al. found that by adding 10 wt.% yttria powder by means of secondary feeding during the APS process, the Y-modified ZSM coatings can actually outperform their virgin counterparts (Ref 280). Furthermore, the structure studied by Liu et al. was in fact a double-layer coating—wherein the base layer was virgin-ZSM, and the topmost layer was the Y-modified ZSM coating. At the same time, Lu et al. published work on a graded ZSM coating, grading the amount of ZrB_2_ and MoSi_2_ through-thickness and showed optimal performance compared to a standard MoSi_2_ coating (Ref 282).

**Fig. 12:**
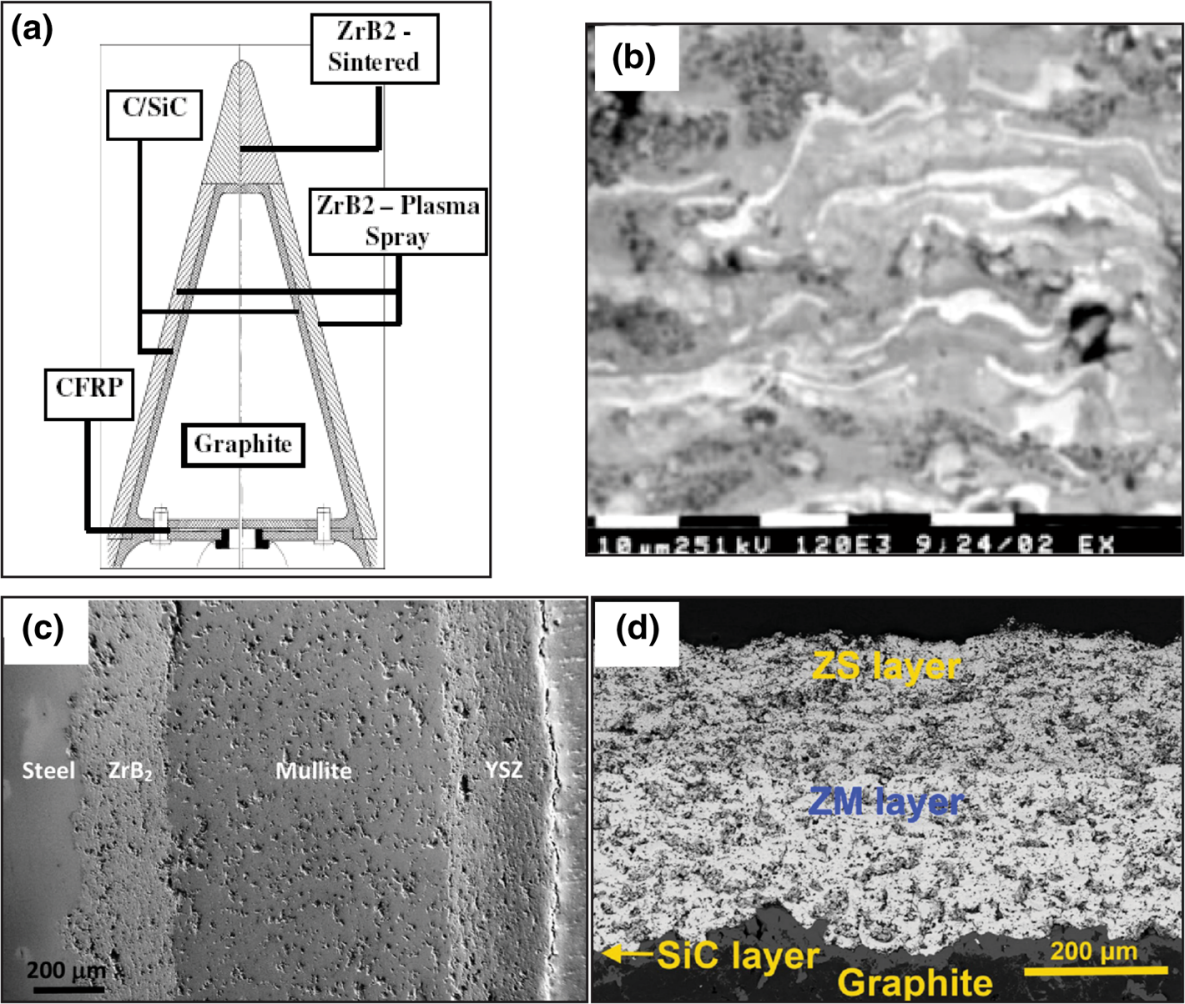
Evolution of UHTC ceramic coatings based on zirconium diboride. (a) The original prototype design for a ZrB_2_ plasma spray coating integrated into a nose cap configuration (Ref 273). Reprinted from Surface and Coatings Technology, Volume 155, Cecilia Bartuli, Teodoro Valente, Mario Tului, Plasma spray deposition and high temperature characterization of ZrB_2_-SiC protective coatings, pp. 260-273, Copyright 2002, with permission from Elsevier. (b) Shows a cross-section from the original CAPS fabricated ZrB_2_-SiC coating from (a) (Ref 274). Used with permission of American Institute of Aeronautics and Astronautics (AIAA), from PRORA-USV SHS: Ultra High Temperature Ceramic Materials for Sharp Hot Structures, L. Scatteia, A. Riccio, G. Rufolo, F. De Filippis, A. Vecchio, G. Marino, AIAA/CIRA 13th International Space Planes and Hypersonics Systems and Technologies Conference, 2005; permission conveyed through Copyright Clearance Center, Inc. (c) Shows a multilayer thermal spray coating design, integrating ZrB_2_ with Mullite and 7YSZ for added thermal protection and enhanced ablation resistance (Ref 278). Reprinted from Vacuum, Volume 88, M.F. Morks, Ivan Cole, Akira Kobayashi, Plasma forming multilayer ceramics for ultra-high temperature application, pp. 134-138, Copyright 2013, with permission from Elsevier. Finally (d) shows the two-layer ZrB_2_-SiC//ZrB_2_-MoSi_2_ coating, which showed marked improvements in ablation resistance due to the integration of an ablative-resistance ZrB_2_-SiC outer layer and a more stable ZrB_2_-MoSi_2_ inner layer (Ref 283). Reprinted from Corrosion Science, Volume 145, Tao Liu, Yaran Niu, Chong Li, Xiaohui Pan, Minhao Shi, Xuebin Zheng, Chuanxian Ding, Ablation resistance of ZrC-MoSi_2_/ZrC-SiC double-layered coating in a plasma flame, pp. 239-248, Copyright 2018, with permission from Elsevier

In 2018, Liu et al. show the advantages and disadvantages of plasma-sprayed ZrB_2_-SiC (ZS) and ZrB_2_-MoSi_2_ (ZM) coatings (Ref 283). In this study, the ZS coating showed delamination after ultrahigh temperature ablation, whereas the ZM coating exhibited the formation of internal bubbles (likely due to the volatilization of SiO_2_ and formation of MoO_3_, as described earlier), which erupted and destroyed the coating. However, Liu proposed and implemented a double-layer solution of ZM followed by ZS, which vastly outperformed either of the two single-layer coatings. The results of the work conducted on UHTC coatings for hypersonics and re-entry applications are summarized in Fig. 12. Clearly, the results shown in this brief overview of the technology demonstrate how the overarching issues in these thermal protection systems stem from materials problems. However, the results in the literature shown here also demonstrate that utilizing multilayer designs is one way to overcome these challenges.

More modern research in the last three years has shown a gradual shift away from the ZrB_2_ system. In its place, HfB_2_-based diborides and ultrahigh temperature carbides (i.e., hafnium and tantalum carbides) have become of interest (Ref 284, 285). These materials are more desirable for modern hypersonics, as they possess even higher melting temperatures and are theorized to have even better ultrahigh temperature ablative resistance than their diboride counterparts. At the moment, the literature in these materials is mainly again focused on developing single-layered solutions, in some cases by using thermal spray technology (Ref 286, 287). However, one can surmise there are similar functional limitations in these new single-layer structures that may be overcome through multilayer engineering.

## Conclusions and Future Outlook

This overview has strived to present several applications and case studies that show the historical evolution of functional thermal spray coatings. This overview has also shown the thermal spray industry is one that can be heavily influenced by the ebb and flow of the global market. A majority of modern thermal spray applications and research efforts are driven toward where the markets are thriving and/or are a known constant—for example aerospace, wear and corrosion control, etc. It can then be argued that the direction of research in thermal spray technology can also be heavily influenced by the directions taken by the industrial market. For instance, the transition from thermal barrier to environmental barrier, and now to thermal-environmental barrier coatings for next-generation turbines, or the revitalization of research in bactericidal coatings for protection of public surfaces in response to the COVID-19 pandemic. A market-driven research approach such as this can be beneficial (i.e., allowing researchers to conduct studies on cutting-edge technology that has real and implicit applications in the field), but this approach also has the potential to hinder growth of the technology as a whole. Some degree of vulnerability in the longevity of the thermal spray market is thereby implied. For instance, if a new materials processing technology were to be discovered that overtakes thermal spray technology in one of its core market shares such as aerospace, this change could cripple the entire thermal spray industry’s financial growth and future expansion.

Given this implied vulnerability, it becomes important to examine alternative market spaces for thermal spray technology, both those that have been relatively established and those that are emerging. In doing so, the goal would be to trace the success over history and use the lessons learned to enable growth in new market spaces. To that end, this overview has presented several case studies of such market spaces wherein thermal spray technology has either seen historical growth but limited adoption, or is just starting to show promise as a feasible materials processing technology for that application. In each of these overviewed application spaces, a historical progression based on the available literature of the technological evolutions in that specific field in the context of thermally sprayed coatings has been presented.

In the cases presented in this overview paper, the level of maturity of implementation and prospective feasibility for thermal spray technology ranges from moderate to advanced. For instance, in the biomedical field(s), there are commercial companies to this day selling plasma-sprayed hydroxyapatite-coated implants for hip replacement surgeries. In the sensor and electronics field, there are companies which have integrated and commercialized thermal spray direct-write technology and provide services to customers for thermally sprayed sensor devices, antennae, etc. However, in other cases, for instance thermally sprayed thermoelectric devices and thermally sprayed gas sensors, the level of technological maturity is significantly lower.

Nevertheless, by studying these diverse applications in this manner, the versatility and flexibility of the thermal spray process have been shown in this overview to present attractive opportunities for new and unique applications. In most cases studied here, the historical progression of research has shown that these thermal spray coatings must have ‘duality in function’—that is to say coatings must serve multiple simultaneous functions where they are being used. In biomedical implants, coatings should have the appropriate characteristics to promote osseointegration, but they must not facilitate the culture and growth of bacterial infections within the implant area. In sensors and electronics, coatings implicitly should have a wide variety of capabilities—for applications such as antennae, humidity sensors, strain gauges, etc. Thermoelectric devices formed by thermal spray manufacturing are envisioned to integrate p-type and n-type discrete layers, and these layers must have intrinsically low thermal conductivities, but simultaneously high electrical conductivities. Gas-sensing coatings must have high specific surface area, but also must possess a gas-selective nature to avoid cross-sensitivity to other undesired gaseous species. Lastly, high-temperature and ultrahigh-temperature coatings for thermal barriers and thermal protection systems must be able to protect the underlying component(s) from extreme incident temperatures and ablative environments, but must also have high toughness, resistance to the surrounding environments, and in some cases must also resist chemical interactions with foreign debris.

These requisite conditions (which often are difficult to achieve simultaneously) imply the need for duality in function. Furthermore, it can be suggested that there is a substantial challenge to find a monolithic coating in any of these overviewed applications that can ‘overcome’ the design requirements. Consequentially, in almost all the application spaces described in this overview, some form of a multilayer, multifunctional coating structure was discussed. This overview has in turn demonstrated the advantages of multilayer thermal spray coatings in various applications to be as follows:

- Multilayer coatings can be engineered and tuned to deliver location-specific properties. Functional surface layers can be integrated with, for instance, adhesion-promoting inner layers, etc.
- Multilayer coatings (have the capability to) utilize a single materials processing technology to realize the structures. For example, in a Multilayer T-EBC, the entire structure from bond coat to topmost layer can be achieved using only air plasma spray.
- Multilayer coatings can be spatially sophisticated as compared to traditional processing counterparts. For example, circuitry and complex patterning can be formed on a surface for mesoscale electronics, sensors, and thermoelectric generators.
- Multilayer coatings can be more cost-effective and durable than their competing systems over long periods. For instance, UHTC multilayers more durably resist ablation in re-entry environments, T-EBC multilayers reduce internal temperatures to enable the use of more robust bond coats, and multilayer TEGs can integrate abundant, low-cost materials for similar performance and output as compared to conventional generators.

Despite the implied advantages, the industry as a whole still sees limited adoption of multilayer thermally sprayed structures. This can be due to the potential apprehension to more complex spraying methodologies, or the implicit increased production costs in adding manufacturing steps, etc. Nevertheless, this overview has intended to utilize this historical approach to overview technological advancements so as to present a framework from which future research in these evolving application spaces can be inspired.
